# Sustainable, Safe, and Effective (Super)Hydrophobic Coatings for Cellulosic Fiber Material via Alkyl Ketene Dimer and Polysaccharide Integration

**DOI:** 10.1021/acssuschemeng.5c08955

**Published:** 2026-02-23

**Authors:** Petra Jerič, Barbara Golja, Gregor Lavrič, Janvit Teržan, Anja Verbič, Blaž Likozar, Uroš Novak

**Affiliations:** † Jožef Stefan International Postgraduate School, Jamova 39, 1000 Ljubljana, Slovenia; ‡ Faculty of Mechanical Engineering, 112807 University of Maribor, Smetanova ulica 17, 2000 Maribor, Slovenia; § Faculty of Natural Sciences and Engineering, 112798 University of Ljubljana, Snežniška ulica 5, 1000 Ljubljana, Slovenia; ∥ Department of Catalysis and Chemical Reaction Engineering, National Institute of Chemistry, Hajdrihova 19, 1000 Ljubljana, Slovenia; ⊥ Pulp and Paper Institute, Bogišićeva 8, 1000 Ljubljana, Slovenia

**Keywords:** water-based hydrophobic coating, cellulosic fiber material, alkyl ketene dimer, polysaccharides, PFAS-free alternative

## Abstract

In light of increasing environmental and regulatory restrictions on per- and polyfluoroalkyl substances (PFAS), silicones, and other persistent synthetic hydrophobic agents, we report the development of novel biodegradable, water-based hydrophobic coatings for cellulosic fiber materials based on alkyl ketene dimer (AKD) and naturally derived polysaccharides alginate, cellulose nanofibers, starch, and agar as matrices. Coatings on the cellulosic fiber material were applied through screen printing and cured at low temperatures. The prepared coatings transformed the initially (super)­hydrophilic cellulosic fibers into a hydrophobic material, with static water contact angles ranging from 126° to 153°. Application of the coatings extended water drop absorption times from immediate uptake to as long as up to 5 h, exhibiting rolling-off behavior consistent with lotus-leaf-like hydrophobicity. SEM–EDX analysis revealed well-defined microstructuring and uniform elemental distribution, confirming complete coverage of the cellulosic fibers. FTIR spectroscopy and sequential organic solvent extraction provided evidence of covalent AKD–cellulose bonding, confirming successful chemical surface modification of the substrate. The coatings demonstrated excellent durability, maintaining hydrophobic performance even after 30 laundering cycles and exhibiting resistance to chemical and mechanical stress. A synergistic effect between AKD and polysaccharides was observed and explained: while AKD imparts intrinsic hydrophobicity, the polysaccharides act as functional stabilizers and physical barriers, improving coating uniformity, adhesion, and long-term performance.

## Graphical abstract

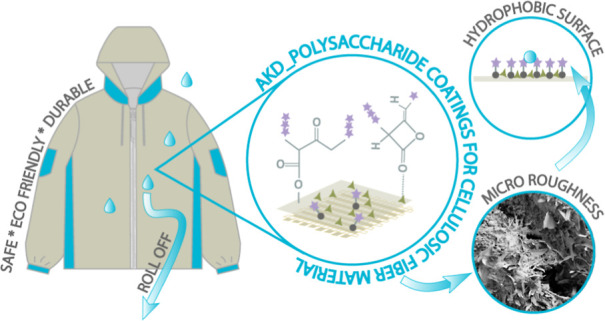

## 1 Introduction

Controlling wetting behavior is increasingly important across various fields, especially in the technical textile industry, where (super)­hydrophobic surfaces enable liquid repellence, self-cleaning, unidirectional liquid transport, and creation of barrier coatings on fiber applications.^1,2^ Current industrial (super)­hydrophobic applications for the cellulosic fiber materials (CFMs) are solely dependent on the per- and polyfluoroalkyl substances (PFAS). However, recent findings have highlighted the need to limit their use, as they pose significant risks to both the environment and human health.^3^ Consequently, new hydrophobic coatings alternatives have been examined such as silicones,^4^ alkylamines,^5^ and acrylates,^6^ can still be toxic and/or nonbiodegradable.^7,8^

Alkyl ketene dimers (AKDs) are traditionally used in paper making as sizing agents to improve tear resistance and prevent ink spreading during writing by enhancing hydrophobicity.^9,10^ Their inherent safety, low-cost, biodegradable,^11^ skin friendly^7^ and highly hydrophobic waxy properties^9^ have been validated by their compliance with the U.S. Food and Drug Administration’s requirements for use in food contact applications.^12^ Most widely used applications cover hydrophobic sizing agents in the paper industry. Furthermore, it has also been used in studies to hydrophobize cellulose^13,14^ and starch composites and films,^9^ forming β-keto ester bonds with the hydroxyl groups as a favorable reaction compared to reacting with water, forming a β-keto acid, which spontaneously decarboxylates to form the corresponding ketone.^15^ The covalent bond formed provides immobilization and proper orientation of the hydrophobic tail away from the surface.^10^

There is growing interest in natural polymers across industries due to their sustainability, biocompatibility, versatility, and low extraction costs.^16,17^ Polysaccharides comprise roughly 63% of the Earth’s total dry biomass and are available in multiple naturally occurring forms that can be isolated at an industrial scale from microbial, plant, and animal feedstocks.^17^ All of them confer hydrophilic properties, enabling them to be easily dispersed or dissolved in water and to form good films.^18^ Moreover, polysaccharides can also function as surfactants and binders, helping to disperse and fix hydrophobic fillers onto surfaces to enhance hydrophobicity, creating a protective layer on textile surfaces and interacting with hydrophobic molecules in the coating. They can improve the coating’s durability, flexibility, adhesion, breathability, and ultraviolet (UV) resistance.^18^ However, their hydrophilic nature can reduce coating performance in humid environments, requiring hydrophobic modifications through chemical or physical treatments·^19^

The AKD reaction mechanism with polysaccharides was found to enhance the hydrophobic properties of starch-based films for food packaging, achieving a water contact angle (WCA of 128°.^9^ AKD treatment of cellulose and nanocellulose films yielded WCA of 103°.^20^ Additionally, AKD combined with chitosan has been used to hydrophobically modify starch-based films for mango preservation,^12^ as well as to create cellulose filter paper through Pickering emulsions—reaching WCAs over 150° using chitosan and titanium dioxide as emulsifiers to disperse particles^21^—and to modify cellulose composite membranes.^13,15^ So far, only a minor focus has been given to the hydrophobization of CFMs using AKD. Onodera et al.^22^ soaked cotton knitted fabrics in weakly cationic aqueous dispersions of AKD, which resulted in prolonged water absorption times (>1800 s), indicating high water repellency even after 30 laundry cycles. Thananukul et al.^23^ developed a method to modify cotton fabrics with water-based AKD nanoparticle dispersions deposited via ultrasonic spraying, achieving a WCA of 137 ± 3°. The treated fabrics maintained high water repellency after 20 washes and UV exposure, demonstrating excellent durability.

AKD–polysaccharide composites have demonstrated biodegradability despite their hydrophobic nature. Wahyuningsih et al.^24^ confirmed that AKD-modified starch biofoams undergo complete fungal degradation within 21 days under a modified ASTM G21 assay, with AKD increasing crystallinity and reducing water uptake, yet not inhibiting microbial colonization. Similarly, Kwon et al.^25^ reported that AKD-treated cellulose fibers remain highly biodegradable (≈77–85% ultimate extent) in ISO 14851 aerobic aquatic tests. While AKD hydrophobization lengthened the lag phase and slowed early biodegradation rates (delays moisture penetration and initial microbial activity) the overall extent of biodegradation was preserved. The growing demand for biobased hydrophobic coatings has brought polysaccharide–fatty acid systems like AKD, maleated high-oleic sunflower oil (MSOHO),^26^ fatty acid anhydrides (e.g., stearic anhydride), epoxidized vegetable oils to the forefront as practical and increasingly sustainable alternatives to conventional textile finishing agents.^27^ The incorporation of polysaccharide stabilizers is often promoted as a means to further improve sustainability by reducing the reliance on synthetic surfactants and enhancing the biodegradability of finishing formulations. When properly formulated, polysaccharide stabilizers can improve the utilization efficiency of fatty-acid-derived hydrophobizing agents, decrease their required dosage, and enable milder processing conditions and waterborne systems—collectively contributing to a more sustainable finishing process.^28^ However, their magnitude depends strongly on the origin and processing of the polysaccharides and on avoiding aggregation-related inefficiencies that can diminish their utilization.^29^

Despite these challenges, AKD–polysaccharide combinations remain one of the few hydrophobic coating systems that couple renewable feedstocks, proven performance, and compatibility with existing processing infrastructure. A critical examination of AKD– polysaccharide systems is therefore essential to identify the conditions under which they deliver true sustainability gains. Given their established industrial stability,^30^ biodegradability, and regulatory advantages over fluorinated chemistries, AKD-based systems continue to represent one of the most realistic and scalable routes toward PFAS-free, biobased hydrophobic textile finishes.

This comprehensive study aims to pioneer the development of truly durable, eco-friendly, and nontoxic (super)­hydrophobic coatings for CFM by combining AKD with natural polysaccharides. A wide range of polysaccharides—alginate, cellulose nanofibers (CNF), starch, and agar—combined with varying concentrations of AKD, applied to CFM, was explored. Evaluation of the coatings included comprehensive surface, chemical, morphological, and physical analyses, along with visual, chemical, abrasion, and washing stability assessments.

## 2 Experimental Section

### 2.1 Materials

Agar (viscosity: 120–150 kDa, estimated DP: 392–490), sodium alginate (viscosity: 80–120 kDa, estimated DP: 370–556), and corn starch (C_6_H_10_O_5_)_*n*_, 106–108 g mol^–1^) were purchased from Sigma-Aldrich (Steinheim, Germany). Cellulose nanofibrils (CNF, 3 wt % gel, −OH, −COOH, unmodified, particle size 6–20 μm, prepared from bleached hardwood kraft pulp, Mw = 214 700 g mol^–1^, 1 wt % lignin, 71 wt % cellulose, 28 wt % hemicellulose, viscosity (1.0% at 20 °C) = 950 mPa·s, pH (1.0%) = 6.9, heavy metals content: Pb < 20 mg kg^–1^, As < 2 mg kg^–1^, total bacterial count <100 cfu g^–1^, total mold and yeast <100 cfu g^–1^, surface charge up to 0.1 mequiv g^–1^) were supplied by Sappi Valida (Maastricht, Netherlands). The AKD dispersion (6–20% 2-oxetanone, 3-C12–16-alkyl-4-C13–17-alkylidene derivatives, 2.5% basic aluminum chloride, <0.0015% mixture of 5-chloro-2-methyl-2H-isothiazol-3-one and 2-methyl-2H-isothiazol-3-one in a 3:1 ratio) was obtained from Melamin (Kočevje, Slovenia). Oeko-Tex Standard 100-certified CFM (weight: 185 g/m^2^) was sourced from Europrint (Slovenia). For testing chemical stability, NaOH (1.0 M) and HCl (1.0 M) were purchased from Kefo (Slovenia). Distilled water was used throughout the experiments.

### 2.2 Preparation of AKD–Polysaccharide Coatings

#### 2.2.1 Experimental Design

Eighteen main coating variations labeled as AKD–polysaccharide coatings were prepared using four polysaccharides: alginate, cellulose in the form of CNF, corn starch, and agar, with AKD applied at three concentrations (1, 5, and 10 wt %). Coatings containing agar and starch were prepared in two ways, by heating and non-heating, to evaluate the effect of possible gelatinization on coating performance. Those main coatings were deposited on CFM.

Additionally, reference formulations were prepared. Coatings without AKD, containing only polysaccharides and water deposited on CFM, were labeled as polysaccharides–only samples. Coatings without polysaccharides, consisting solely of AKD and water deposited on CFM, were labeled as AKD–only samples. Coating samples in the form of films, without deposition on CFM, were also included and labeled as AKD–polysaccharide coating–only samples (COS). The reference coating samples enabled a clearer assessment of the individual contributions of each component to the overall coating performance. An overview of all prepared samples, their formulations, and the labels used throughout this article is presented in Figure 1. A substrate–only sample (untreated CFM) was also tested and analyzed for comparison.

**Figure 1:**
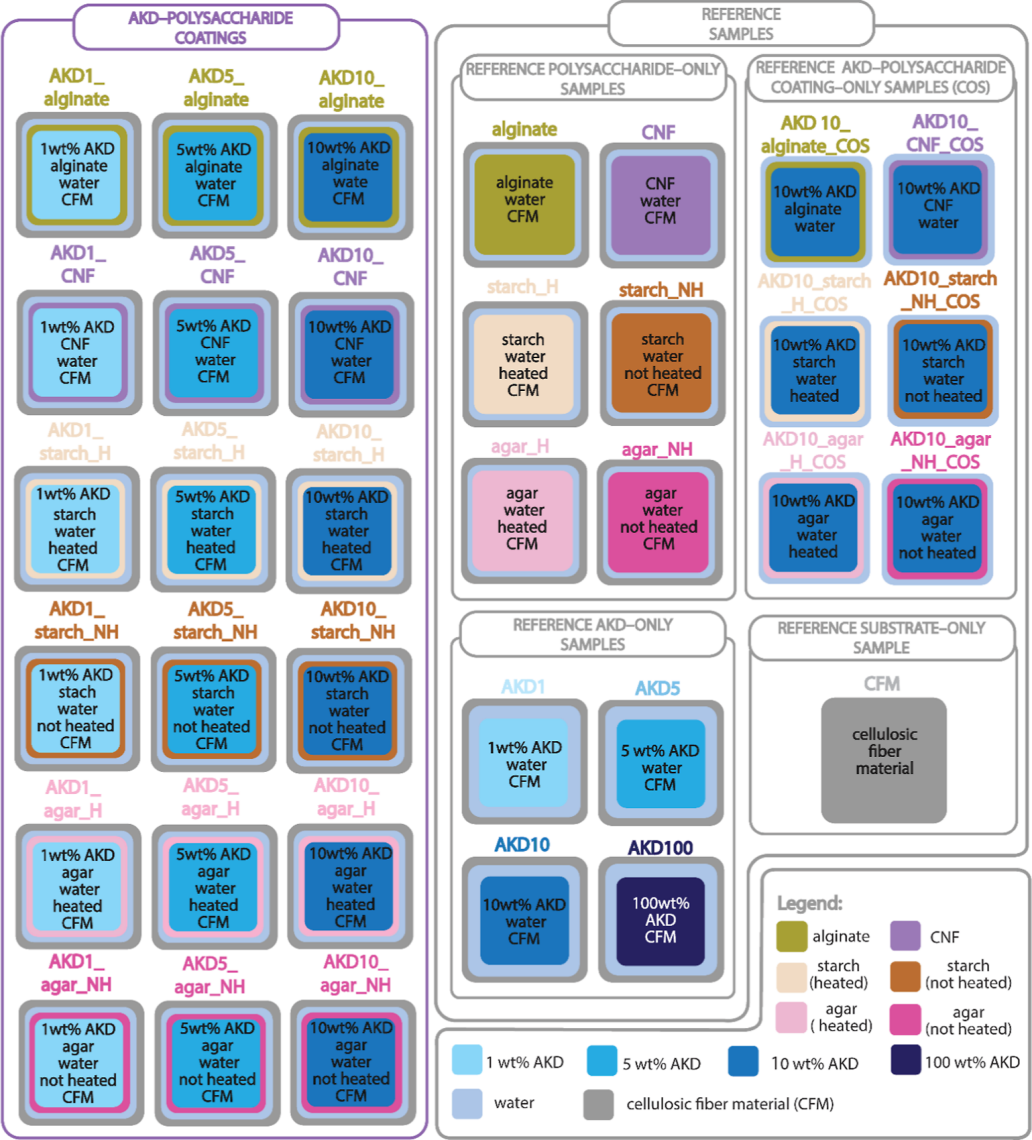
Experimental design illustrating all tested samples and their labeling, including the exact components of each system. The main sample group comprises AKD–polysaccharide coatings deposited on cellulosic fiber material (CFM). Reference samples include polysaccharide–only, AKD–only, and AKD–polysaccharide coating–only samples (COS), as well as the substrate–only CFM sample.

#### 2.2.2 Preparation of the Coatings before Deposition

The preparation of the coatings is schematically presented in Figure 2. First, AKD, polysaccharide, and distilled water were mixed using a blender at 2000 rpm for 1 min to obtain a homogeneous solution. For coatings that did not require heating, the mixture was directly applied to the CFM. Coatings requiring heating were placed on a heating plate with a magnetic stirrer and heated at 100 °C for 10 min, allowing the mixture to form a gel-like consistency before application.

**Figure 2:**
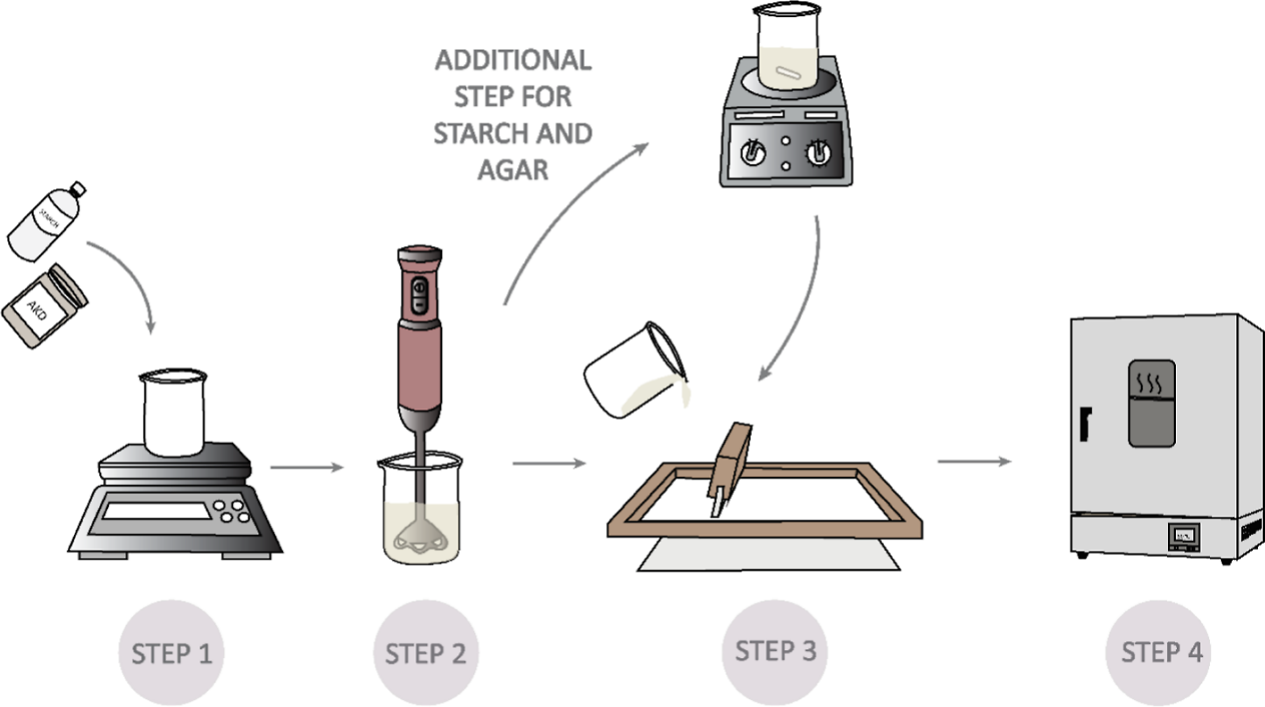
Schematic overview of the sample preparation procedure, comprising weighing of components, formulation mixing, additional heating for AKD_starch and AKD_agar systems, screen-printing deposition onto cellulosic fiber material (CFM), and thermal curing in a laboratory oven.

#### 2.2.3 Deposition of the Coatings and Curing Treatment

CFM was cut into 35 × 20 cm rectangles in both the warp and weft directions and placed on a flat surface. A screen was then positioned over the fabric, and 50 mL of the coating solution was poured onto the screen. The coating was evenly spread using a squeegee. Following this step, each sample was placed in a laboratory oven at 60 °C for 20 min. The optimal curing conditions were identified by systematically drying the samples across a range of temperatures (room temperature, 40 °C, 50 °C, 60 °C, 70 °C, 80 °C, 90 °C, 100 °C, 110 °C, 120 °C, and 130 °C) and curing times (no curing, 5, 10, 15, 20, 30, 40, 50, 60, 70, 80, 90, 100, 110, 120, and 130 min). The final curing temperature and time were selected based on these experimental results, with additional consideration given to minimizing electricity consumption.

### 2.3 Characterization of AKD–Polysaccharide Coatings

#### 2.3.1 Attenuated Total Reflectance- Fourier Transform Infrared Spectroscopy (ATR-FTIR)

ATR-FTIR was conducted to identify the functional groups present on the coating surface, with a particular focus on those associated with its hydrophobic properties. Infrared spectra were measured using a PerkinElmer Spectrum GX spectrometer (Waltham, MA; USA). The spectra were obtained under standard conditions within the range from 4000 to 400 cm^–1^, using a resolution of 2 cm^–1^. Each spectrum was averaged from 16 scans and had background spectra subtracted. At least 2 measurements were performed for each sample. For data analysis Origin software was used. To facilitate comparison, normalized data were used for analysis.

#### 2.3.2 X-ray Photoelectron Spectroscopy

XPS was performed to determine the different carbon species on the samples. The analysis was performed with the PHI VersaProbe 3 AD (Phi, Chanhassen, US), which uses a monochromatic Al Kα X-ray source. The charging of the sample was attenuated with electron and ion beams. The peak shift caused by the neutralization was corrected by shifting the peaks according to the Au standard measured immediately after the samples. The survey spectra were measured at a transit energy of 224 eV with a step of 0.8 eV. The high-resolution spectra were measured with a transit energy of 27 eV and a step size of 0.05 eV. For the survey spectra, 2 sweeps were performed, while for the high-resolution spectra, 20 sweeps were performed. The spectral deconvolution was performed with the KherveFitting software.

#### 2.3.3 Scanning Electron Microscopy–Energy Dispersive X-ray Spectroscopy (SEM–EDX)

A small piece (≅1 × 0.8 cm) of each sample was set onto carbon tape. The morphology of the samples was examined under vacuum conditions using a SEM SUPRA 35VP (Carl Zeiss, Jena, Germany) using a 1 kV electron beam accelerating voltage and 4.5 mm working distance. Prior to analysis, the samples were coated with a 6 nm layer of gold to enhance conductivity and minimize charging effects. The samples were examined and analyzed at 150×, 1000×, and 10 000× magnifications. EDX analysis was performed with an electron high-tension voltage of 10.0 kV and 8.5 mm working distance.

#### 2.3.4 Geometrical Surface Roughness

Geometrical surface roughness was evaluated using the Kawabata Evaluation System for Fabrics: KES-FB4 surface tester. Samples were cut into 20 × 20 cm squares and mounted between two specially designed clamps. One clamp was embedded in the surface of a rotating delivery drum and secured the specimen using a metal pin. The second clamp, constructed as a spring-loaded clip, was orthogonally fixed to a movable arm, which applied a constant pretension force equivalent to a 400 g weight. This setup enabled smooth translation of the fabric between a reference metal plate and the measuring probe, which simulates the tactile interaction of human fingertips. During testing, a constant normal load of 10 g was applied by the sensing head onto the specimen. The fabric was moved steadily by the delivery drum, and the system’s built-in potentiometer recorded directional changes and surface response. Measurements were conducted in both warp and weft directions, with three repetitions performed for each orientation to ensure repeatability and minimize variability.

#### 2.3.5 Water Contact Angle Measurements

To measure the WCA of the samples, the sessile drop technique and the OCA 35 goniometer (Data Physics, Germany) were employed. Samples were cut into 3 × 0.5 cm^2^. The samples were placed on the apparatus’s solid plate below a stainless-steel needle with an inner diameter of 0.16 mm. A 3 μL droplet of Milli Q ultrapure water was automatically dispensed, forming a droplet on the sample surface. The contact angle was determined using the Laplace–Young equation. Each sample was tested with at least five replicates and results are represented as a mean value with standard deviations. WCAs were measured on samples stored under varying ambient room conditions (20–25 °C, 30–60% RH) as well as on samples stored for 48 h in a controlled laboratory climate chamber (20 °C, 65% RH).

#### 2.3.6 Gravimetrical Analysis

Gravimetric analysis was employed to determine the percentage of coating deposited on the CFM samples using an analytical weighing scale (KERN, Germany). Each sample was weighed before and after coating application. The mass difference was attributed to the coating layer, and the coating percentage was calculated relative to the total mass of the coated sample. In addition, the method was utilized during stability tests to detect any changes in sample mass, which could indicate degradation or loss of the coating material.

#### 2.3.7 Surface Tension

The surface tension of the coating dispersions was measured using a Wilhelmy plate with known dimensions, following DIN 53 914, and a K12 tensiometer (KRÜSS GmbH, Germany). During the measurement, the drive table holding the tested solution (100 mL) was raised until it made contact with the bottom edge of the platinum plate. The force measured was proportional to the surface tension and the contact angle between the solution and the platinum plate. The K12 Wilhelmy plate setup software automatically determined the surface tension by performing a regression analysis on the measurement data points. Each measurement was repeated three times. The average value and standard deviation were calculated for the measurements obtained.

#### 2.3.8 Surface Zeta Potential

The streaming potential measurements were conducted using a SurPASS 3 (Anton Paar GmbH, Austria) at room temperature. Samples were cut to dimensions of 20 mm × 10 mm. Each sample pair was affixed to the sample holder using double-sided adhesive tape. The distance between the sample surfaces was set to 110 ± 10 μm. The surface zeta potential was measured as a function of pH in an aqueous electrolyte solution of 10 mM KCl. The pH was automatically adjusted using 0.05 M KOH and 0.05 M HCl. Before measurement, the solid sample was equilibrated at a neutral pH with several rinsing steps, followed by adjustment to the alkaline range. A pressure gradient of 200–600 mbar was applied to generate the streaming potential, which was measured using a pair of AgCl electrodes. The pH and conductivity of the electrolyte were continuously monitored with pH and conductivity probes.

#### 2.3.9 Air Permeability

Air permeability was assessed using a Karl Schröder apparatus (Karl Schröder KG, Weinheim, Germany) in accordance with the ISO 9237 standard. The sample measurement area was 20 cm^2^. Each test sample was placed under tension and positioned over the clamping head. The air flow regulator was set to its maximum value of 1, and the corresponding scale value was recorded. At least three measurements were taken for each sample. The air permeability of the coated CFM samples was then calculated using the eq 1

1 $$VN=f\times VG\times \sqrt{\frac{PU\times TN}{PN\times TU}}$$

VN is the reference air permeability [L/m^2^/s], *f* is the conversion factor for the used clamping surface area (1 for 20 cm^2^), VG is the measured air permeability [L/m^2^/s], PU is the measured atmospheric pressure [mbar], TN is the reference temperature (293 K), PN is the reference atmospheric pressure (1013 mbar), TU is the measured temperature [K].

#### 2.3.10 Mechanical Properties

The mechanical properties of the samples, including tensile strength and elongation at break, were assessed using a Zwick Roell Z010 tensile testing machine (Zwick Roell, Ulm, Germany) in accordance with the DIN EN ISO 13934-1:2013 standard. Test specimens, measuring 5 × 25 cm, were prepared with samples cut in both the warp and weft directions. Prior to testing, the thickness of each sample was measured 10 times using a precision thickness gauge, with the average thickness value used in subsequent mechanical property evaluations. Testing was conducted under controlled conditions at 23 °C and 50% relative humidity. The crosshead speed was maintained at 20 mm/min, with a clamp distance of 20 cm. During testing, load and elongation data were captured multiple times per second until the sample reached failure.

#### 2.3.11 Abrasion Resistance

The abrasion resistance of the fabrics was evaluated using the Martindale method according to the SIST EN ISO 12947-2:2017 standard. The test specimens had a diameter of 38 mm, and the abradant used was woven wool felt. The total effective mass of the abrasion load was 595 ± 7 g, corresponding to a nominal pressure of 9 kPa. Samples were visually inspected and subjected to WCA measurements after 5 000, 10 000, 15 000, and 20 000 cycles. All measurements were conducted at a temperature of 20 °C.

#### 2.3.12 Chemical Stability

The methodology for testing chemical stability was adapted from procedures outlined in relevant research literature.^31^ Solutions with acidic, alkali, and neutral pH levels were prepared using a Mettler Toledo pH meter. An acidic solution (pH = 1,5) was created by adding HCl to distilled water, a neutral solution (pH = 7) was prepared using only distilled water, and alkali solution (pH = 12,5) was made by adding NaOH to distilled water. Coated CFM samples, cut into 4 cm^2^ squares, were immersed in each of the three solutions, with three samples of each coating type used for every solution. The weight of each sample was measured before and after immersion to assess any coating dissolution. Observations were made at three intervals: 1, 24, and 48 h. After the treatment, the samples were air-dried. WCAs were measured on the dried samples.

#### 2.3.13 Washing Stability

The samples were washed using a Gyrowash 815 (James Heal, UK) following the ISO 105C06 standard. For each sample, three replicates measuring 10 × 4 cm were cut. Each washing cycle was conducted at 40 °C for 45 min in a 150 mL of optical brighteners free ECE standard detergent solution (4g/l), with the addition of 10 steel balls One such washing is equivalent to 5 domestic washings. In the case of 10, 20 or more washings, the procedure was appropriately repeated several times with intermediate air drying of the samples. WCA values were measured post-drying drying as described in Section 2.3.5 Water contact angle (WCA) measurements. Washing stability was observed after 5, 10, 20, and 30 washing cycles.

#### 2.3.14 Sequential Extraction of the Coating Compounds with an Organic Solvent

Unbound AKD was removed from the coated samples by sequential extraction with hexane. Each 3 × 3 cm fabric specimen was immersed in 50 mL of hexane in a clean glass beaker. The beakers were placed in a water bath and ultrasonicated for 4 h using an Elma Transsonic T 820H ultrasonic device. To ensure complete removal of unbound AKD, the extraction procedure was performed twice.

## 3 Results and Discussion

The primary objective of the study was to validate the effective integration of AKD with natural polysaccharides as a coating on CFM. By combining AKD—a widely used hydrophobic agent—with renewable polysaccharides, we aimed to develop a coating that retains robust hydrophobic functionality while enhancing the overall sustainability of the treatment and could be potentially used for different textile applications. The characterization methods focused on confirming the successful deposition of the AKD–polysaccharide coatings on CFM, gaining insight into the underlying chemical reaction mechanisms between AKD and both polysaccharides and cellulose in the substrate, and verifying the expected hydrophobic properties of the coatings. It was also investigated how different types of polysaccharides affect the coating behavior and examined the influence of varying AKD concentrations on the efficiency and long-term performance of hydrophobic treatment. Additionally, the effect of the coating on the textile’s mechanical properties and its feel against the skin was evaluated and compared to that of untreated CFM.

### 3.1 Surface Chemistry of AKD–Polysaccharide Coatings and Their Interactions with CFM

To better understand the AKD–polysaccharide interaction with the CFM, first, a FTIR spectroscopy was conducted to observe the changes in chemical structure after the coating deposition. Spectra of untreated CFM sample, pure AKD, AKD–polysaccharide coatings, and reference AKD–polysaccharide COS were analyzed and compared (Figures 4, S1 in Supporting Information). The untreated sample surface spectra exhibited typical functional groups, including the intense band of hydroxyl groups at 3339 cm^–1^ and 2868 cm^–1^, which makes the surface superhydrophilic. We can also notice alkyl C–H stretching at 2898 cm^–1^, and C–H bending at 1425 and 1318 cm^–1^. The most subtle chemical changes were detected in the AKD_agar_NH samples, where only a slight increase in the AKD characterization peak region between 2971–2851 cm^–1^ (area III in Figure S1) was observed. In contrast, other AKD–polysaccharide coatings showed more pronounced peaks, indicating greater asymmetric C–H stretching and suggesting the presence of a more hydrophobic surface. The increase in intensity of characteristic peaks with higher AKD concentrations indicates greater incorporation of AKD into the coating. Additionally, noticeable shifts in the peak positions when comparing AKD–polysaccharide coatings versus reference AKD–polysaccharide COS suggest the occurrence of chemical or physical interactions between the coating components and the cellulosic substrate. The O–H stretching region (3200–3500 cm^–1^; area II in Figures 4a, S1), characteristic of hydrogen-bonded hydroxyl groups in cellulose and other polysaccharides, shows a marked decrease in intensity for both the AKD–polysaccharide coatings and reference AKD–polysaccharide COS deposited on the CFM relative to untreated CFM. This reduction is consistent with the formation of a more nonpolar hydrophobic surface layer and indicates that a fraction of the accessible O–H groups is either involved in new hydrogen-bonding interactions or chemically consumed during the reaction with AKD. This interpretation is further supported by the EDX results (Figures 4b, S2), which show that the coated samples exhibit an increased carbon content—correlating with the enhanced C–H peak observed in FTIR—and a corresponding decrease in oxygen content, consistent with a hydrocarbon-rich AKD layer covering the cellulose surface. This is supported by XPS analysis (Figure 4c), where the peak at 284.8 eV shows a pronounced increase in the C–C signal intensity upon treatment, while the C–O signal at 286.4 eV exhibits a substantial decrease. A new peak corresponding to free O–H stretching vibrations at around 3700 cm^–1^ (area I, Figures 4a, S1), observed only for AKD–polysaccharide coatings deposited on CFM, provides further evidence of chemical changes. This peak reflects newly accessible surface O–H groups that become exposed, confirming that the CFM–polysaccharides–AKD interface generates reactive hydroxyl sites available for covalent bonding.

The peaks observed exclusively in the pure AKD and reference AKD–polysaccharide COS at approximately 1800 cm^–1^ and 1670 cm^–1^ (area IV, Figures 4, S1) can be assigned to the stretching vibrations of the carbonyl (C=O) group and the alkene (C=C) bond in the lactone ring of AKD, respectively. The presence of these characteristic peaks indicates that the AKD lactone ring remained intact and did not undergo chemical opening or transformation. Moreover, the absence of any peak shifts or intensity changes in this spectral region between the pure AKD and reference AKD–polysaccharide COS suggests that no chemical reaction occurred between AKD and the polysaccharides within the coating. When the AKD–polysaccharide coating is applied to CFM, the bands in this region show slight shifts and reduced intensity, indicating that the reactive functional groups of AKD are partially consumed during chemical transformation, most likely esterification or a nucleophilic ring–opening reaction with hydroxyl groups present on the cellulose surface already reported elsewhere.^9,10^ The dominant carbonyl band appears at ∼1723 cm^–1^, consistent with ester/β-ketoester formation resulting from the reaction of AKD with accessible cellulose −OH groups. Additionally, a smaller high-frequency peak near 1850 cm^–1^ reflects a minor population of unreacted or differently oriented strained carbonyl species. In the coated samples, the β-ketoester carbonyl bands are only weakly visible or even absent. This does not necessarily imply a lack of reaction. Rather, β-ketoester signals are often difficult to resolve due to the low degree of esterification relative to the total cellulose −OH population, strong overlap with native cellulose absorptions in the 1660–1750 cm^–1^ region, and the shallow surface penetration depth of ATR-FTIR.^32^ Because most ester bonds form at the buried AKD–cellulose interface, they fall broadly outside the adequate sampling depth of the ATR crystal. Furthermore, β-ketoesters may exist in multiple tautomeric forms, broadening and weakening their carbonyl absorptions.^33–35^ To further confirm the presence of irreversible chemical linkages, additional tests were performed. After sequential extraction with hexane, the samples remained hydrophobic (WCA ∼135°) and continued to display β-ketoester signals in the FTIR spectra, even after prolonged extraction (Figure S3a,b). The mass loss before and after extraction was 0.02%, indicating that most of the removable, nonbound coating was successfully extracted, while the chemically bonded fraction remained attached to the fibers. The chemical reaction is further supported by XPS results, which show an ester/ketone C=O absorption peak at 287.9 eV, although only a small amount of these linkages is present. The absence of intense ester signals may also be attributed to the limited extent of β-keto ester formation by commercial AKD, as typically only 15–40% of the AKD molecules remain immobile until the particles melt due to their high shape stability.^36^ Proposed chemical mechanism is shown in Figure 3.

**Figure 3:**
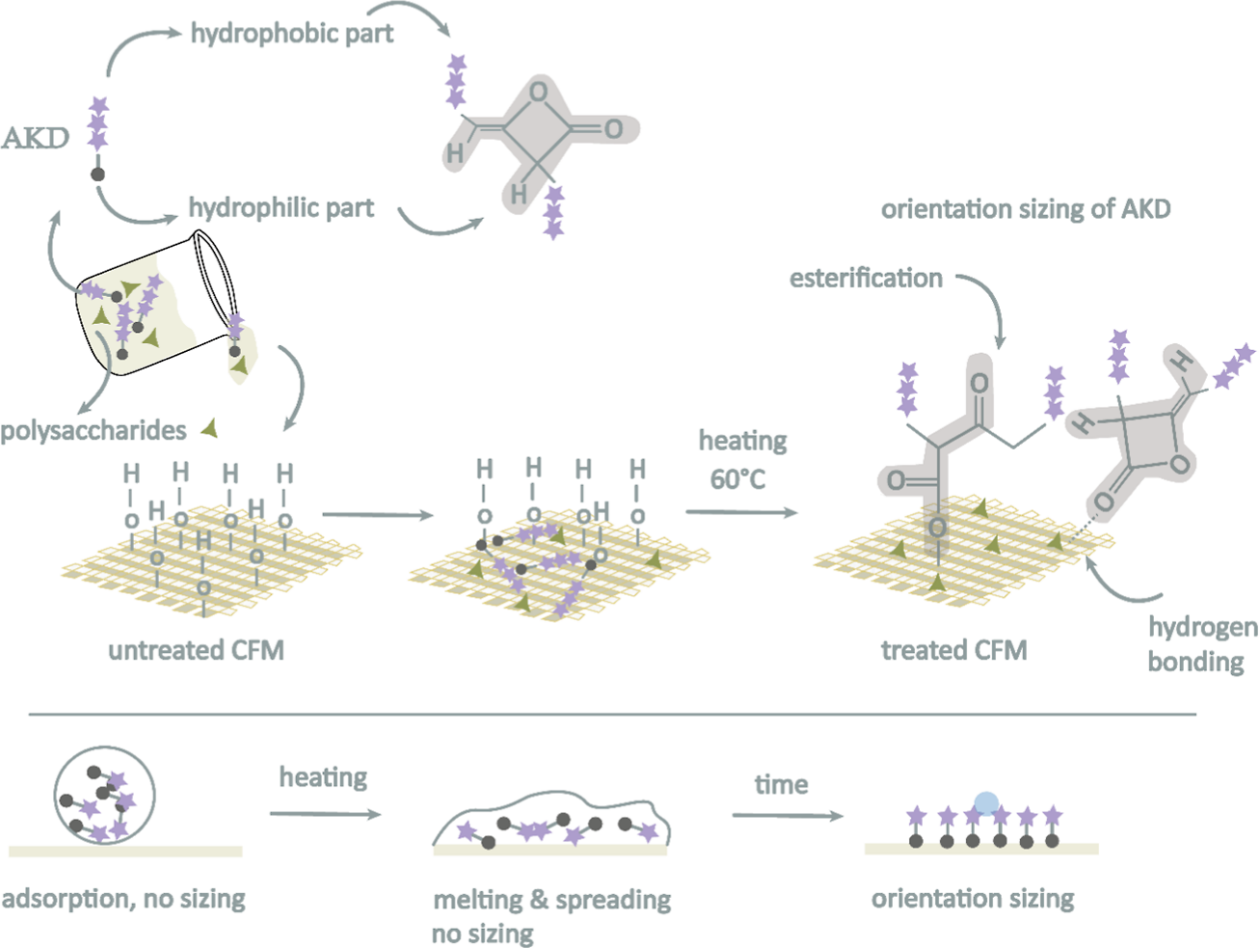
Schematic representation of the proposed chemical mechanism of AKD–polysaccharide coatings deposited on cellulosic fiber material (CFM). The reactive ketene (lactone) ring of AKD reacts with hydroxyl groups of cellulose to form β-keto ester bonds, while simultaneous physical interactions (hydrogen bonding) also occur. The resulting covalent linkage immobilizes the AKD molecule and promotes proper orientation of the hydrophobic alkyl chains away from the surface.

Zeta potential measurements (Figure 4d) of coatings on CFM surfaces provided critical insights into the interfacial chemical environment and extent of surface functionalization. The coatings exhibited markedly different electrokinetic behaviors, strongly influenced by both the nature of the polysaccharide matrix and the AKD concentration. Coatings containing alginate—known for its abundant carboxylate functionalities—displayed the most negative surface potentials (reaching −28 mV). This effect persisted even in the presence of AKD, which is closely associated with the anionic nature of alginate. This suggests that while AKD may partially react with or physically mask some functional groups, a significant fraction of ionizable carboxyl groups remains accessible at the interface, resulting in strong electrostatic contributions to the surface charge. This is further supported by the pH-dependent trend, where zeta potential sharply decreases at higher pH, consistent with progressive deprotonation^37,38^ of weakly acidic −COOH groups. In contrast, coatings formed with CNF, agar, and starch exhibited significantly less negative zeta potentials, especially at higher AKD loadings. This behavior is likely due to a combination of lower inherent acidity and more extensive surface coverage by hydrophobic AKD chains, which not only reduce the net polarity of the surface but also spatially hinder the exposure of polar groups to the aqueous environment during measurement.^9^ The nearly neutral or slightly positive values may also hint at preferential orientation or aggregation of AKD molecules during film formation, resulting in outward-facing nonpolar domains^28^ as shown in Figure 3. Significantly, all coated systems deviated from the zeta potential of native cellulosic fabric material (∼−12 mV), are reflecting successful surface modification. The shift in zeta potential correlates with the mechanism of the reactive β-lactone moiety with surface −OH groups, leading to covalent anchoring of long-chain alkyl groups and suppression of surface polarity.^28^ Overall low zeta potential means that the hydrophilic groups (e.g., −OH) are covered or chemically bound (with AKD), so that water does not interact with them. Increasing AKD concentration amplified this effect, progressively attenuating the magnitude of surface charge as hydrophobic domains became more dominant and ionizable groups became less accessible or chemically consumed.

**Figure 4:**
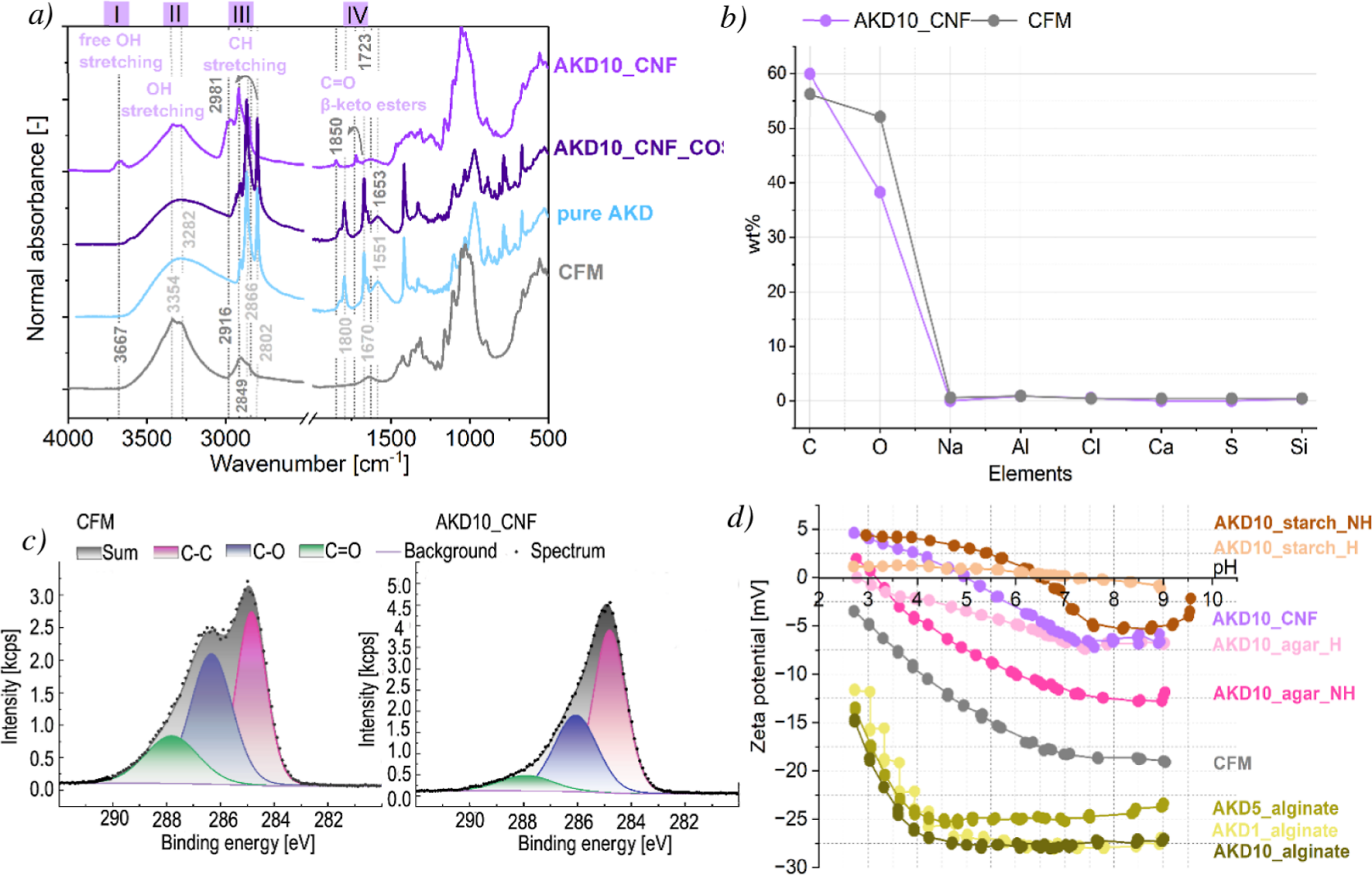
(a) FTIR spectra of untreated cellulosic fiber material (CFM), pure AKD, AKD–polysaccharide coating–only sample (AKD10_COS), and AKD–polysaccharide coating (AKD10_CNF), showing increased C–H stretching bands and the appearance of ester/β-ketoester-related bands after coating. (b) EDX elemental analysis of untreated CFM and AKD10_CNF, indicating an increased carbon content and a decreased oxygen content upon coating. (c) XPS spectra of untreated CFM and AKD10_CNF, showing an increase in the C–C signal intensity and a decrease in the C–O signal after surface modification. (d) Zeta potential of untreated CFM and AKD–polysaccharide coatings, demonstrating a shift relative to untreated CFM and confirming successful surface modification.

### 3.2 AKD–Polysaccharide Coating Surface Properties

#### 3.2.1 The Influence of Curing Parameters on the Hydrophobicity of Coated Samples

The curing temperature plays a crucial role: upon heating, AKD spreads to minimize surface energy, as the cellulose–air interface has a higher surface energy than the air–AKD interface.

In its molten state, the reactive lactone ring of AKD forms stable β-ketoester bonds with cellulose hydroxyl groups, while its nonpolar alkyl chains orient toward the surface, thereby enhancing hydrophobicity^23^ as shown in Figure 3. The aim of this analysis was to identify curing conditions that yield an efficient and durable coating, while simultaneously optimizing the process from a sustainability perspective.

As shown in Figure 6d, drying at room temperature (∼23 °C) yields a hydrophilic WCA, confirming that a continuous hydrophobic surface film does not form under these conditions. AKD remains in a solid, crystalline state with minimal molecular mobility, preventing the spreading or reorientation required to establish a uniform hydrophobic layer. Despite the absence of surface hydrophobization, the FTIR spectra (Figure S4)—reflecting bulk material composition—still show increased C–H stretching, reduced O–H stretching, and the appearance of weak β-keto ester bands. This indicates that limited β-keto ester formation between AKD and cellulose can occur below the AKD melting point, which also previously observed elsewhere.^39^ However, the reaction progresses very slowly and only at localized contact points between AKD particles and the cellulose surface, and leaves most of the cellulose surface unprotected. This limited, point-like covalent bonding may hinder AKD chain mobility by anchoring particles in place,^22,39^ further restricting their ability to spread and coalesce into a uniform hydrophobic layer. At higher temperatures (40 °C), the substrates become hydrophobic. As the curing temperature approaches ∼60 °C, a sharp increase in WCA to approximately 140° is observed. Such behavior is consistent with the known AKD melting and rearrangement processes, which typically begin around 40–60 °C depending on chain length, purity, and formulation.^39^ At this temperature, the β-keto ester peaks show the highest intensity, indicating the greatest extent of covalent AKD–cellulose bond formation. Combined with the highest measured WCA, this temperature was therefore selected as the optimal curing condition. Although FTIR trends suggest that curing at 70 or 80 °C may yield slightly higher carbonyl intensities, the differences in WCA are minimal; thus, 60 °C was selected due to its substantially lower environmental impact. At higher curing temperatures (≥100–130 °C), the WCA reaches a slight plateau, indicating that the surface is already saturated with rearranged AKD and that further heating does not improve molecular packing or the extent of reaction.^39^ Next to this, these temperatures also align with the onset of thermal degradation in cellulose.^40^ The WCA and FTIR data together indicate that thermal energy primarily accelerates the physical redistribution and spreading of AKD rather than significantly increasing its chemical conversion. In this system, the uniform distribution of AKD across the surface plays a more dominant role than molecular orientation in determining the resulting WCA.

Curing time is another critical kinetic parameter that dictates the final hydrophobic performance and durability of AKD-treated CFMs, particularly when utilizing a curing temperature of 60 °C. At very short curing durations (<15 min), the latent sizing agent does not achieve sufficient thermal mobilization or distribution, resulting in incomplete surface coverage and consequently low WCA values. Conversely, curing times extending between approximately 15 and 40 min define the optimal kinetic window, during which WCA dramatically increases to its maximum stable value, typically between 130° and 140°. This rapid transition confirms the efficient physical reorganization, characterized by the melting and spreading of AKD) across the hydrophilic substrate. This ideal molecular arrangement—where the hydrophobic alkyl chains are oriented optimally outward—is substantiated by time-resolved FTIR spectroscopy (Figure S5). The completion of this structural rearrangement phase coincides with the formation of durable beta-keto ester covalent bonds. Extension of the curing process beyond 40 min offers no meaningful gain in hydrophobicity, as surface saturation and bonding equilibrium are already achieved. The literature indicates substantial variation in curing times and temperatures, with reported values spanning from 45 to 120 °C.^10,21,22,28^ Higher temperatures can be connected with shorter times,^22^ but not necessarily.^28^ Our trends in curing parameters correspond well with observations in some other studies, where adequate AKD curing typically occurs within 50–80 °C and 20–40 min^21,39^

#### 3.2.2 Influence of Formulation, Storage Conditions, and Aging on the Hydrophobicity of Coated Samples

The WCAs of AKD–polysaccharide coatings were measured after storage under both ambient and controlled conditions (Figure S4), as it is known that polysaccharides and AKD can undergo changes in functionality when exposed to uncontrolled environmental factors.^9,10,33,41^

As expected, reference polysaccharide–only samples did not increase the WCA of the inherently hydrophilic cellulosic substrate. In contrast, reference AKD–only coatings confirmed its role as the active hydrophobizing agent, achieving WCAs of 152°, 153°, 155°, and 160° for AKD_1, AKD_5, AKD_10, and AKD_100, respectively (Figures 6a, S6c in Supporting Information). Across all formulations, samples stored in a climate chamber showed WCAs up to 17° higher than those stored under ambient conditions, highlighting the strong sensitivity of AKD-based hydrophobization to humidity and temperature fluctuations. This agrees with literature reporting that uncontrolled environmental conditions promote AKD hydrolysis and disrupt migration and crystallization at the solid–air interface.^33^ Weak hydrogen bonding between AKD’s carbonyl groups and polysaccharide hydroxyls can only temporarily anchor AKD, but thermal energy (even at ambient temperature 23 °C) allows eventual detachment, diffusion, and restructuring, or moisture-driven swelling of the biopolymer matrix, all of which can contribute to reduced apparent hydrophobicity when stored in uncontrolled environments.^10^

AKD–polysaccharide coatings yielded WCAs between 126° and 144° under ambient storage, except for AKD1_agar_H (hydrophilic) and AKD5_agar_H (110°). Controlled storage improved these values substantially, with AKD1_agar_H and AKD5_agar_H reaching 127° and 137°, respectively. The highest superhydrophobic WCA of 153° was observed for the AKD10_CNF sample. The differences in WCA values among the AKD polysaccharide coatings at identical AKD concentrations indicate that the intrinsic properties of each polysaccharide play a key role in coating efficiency, primarily by influencing the rearrangement, migration, and overall distribution of AKD molecules during film formation. Those characteristics are hydroxyl group density and accessibility, network porosity and pore size distribution, chain flexibility and segmental mobility, hydrophilic/hydrophobic balance, crystallinity, and ordered domains.^9,10,42,43^ Those specific characteristics of each polysaccharide are represented in the next section.

Polysaccharides heated prior to deposition produced lower WCAs, likely due to premature AKD–polysaccharide esterification. Once covalently bound, AKD loses its ability to migrate and form a continuous hydrophobic layer, resulting in reduced performance. Increasing AKD concentration led to modest increases in WCA (typically within 10°), with the highest value (153°) obtained for AKD10_CNF (Figures 6a, S6b).

Hand in hand with the influence of storage conditions is the effect of aging (Figure 6b). Samples directly after the coating was applied, particularly AKD1_agar_H and AKD5_agar_H, exhibited unexpectedly low WCAs immediately after curing but became hydrophobic after 60 days. This phenomenon reflects spatial time-dependent redistribution rather than molecular reorientation, as spectroscopic studies have shown that AKD hydrocarbon chain orientation remains largely invariant once deposited.^39^ Similarly to our observation, Adenekan and Hutton-Prager^44^ reported that AKD-coated samples require approximately 20 days to develop significant hydrophobicity, with surfaces reaching stable and durable performance after 40 days. While Li and Neivandt^39^ reported similar progressive increases.

The behavior of the water droplet was observed through absorption time (the time until the water droplet is fully absorbed into the fabric) and a practical pouring test. The water absorption time for most AKD–polysaccharide coatings was approximately 5 h. Exceptions include the AKD1_agar_NH sample, which absorbed the droplet within 30 min, and the AKD1_CNF sample, whichabsorbed it after 3 h. Visual observations of water drop absorption immediately after deposition and after 1 h are shown in Figures 5c,d, and S7a,b, demonstrating the excellent hydrophobic behavior of the AKD–polysaccharide treated CFM. Notably, the water droplet on the AKD1_agar_NH sample was absorbed along with a diffuse wetting stain, exhibiting behavior similar to untreated CFM. In contrast, water droplets on the other samples produced smaller, well-defined drop-shaped marks, indicating a slower absorption rate and more effective hydrophobic modification. It was also observed that water droplets did not adhere to the surface of the treated fabric when the water was poured on the fabric or the fabric was tilted; instead, they rolled off easily. Figure 5a,b show the behavior of the untreated and treated CFM garments after colored water was poured onto them. As shown, the untreated CFM absorbs the water, resulting in a large stain. In contrast, the treated fabric repels water during pouring, and the droplets roll off the surface, leaving only minimal marks.

**Figure 5:**
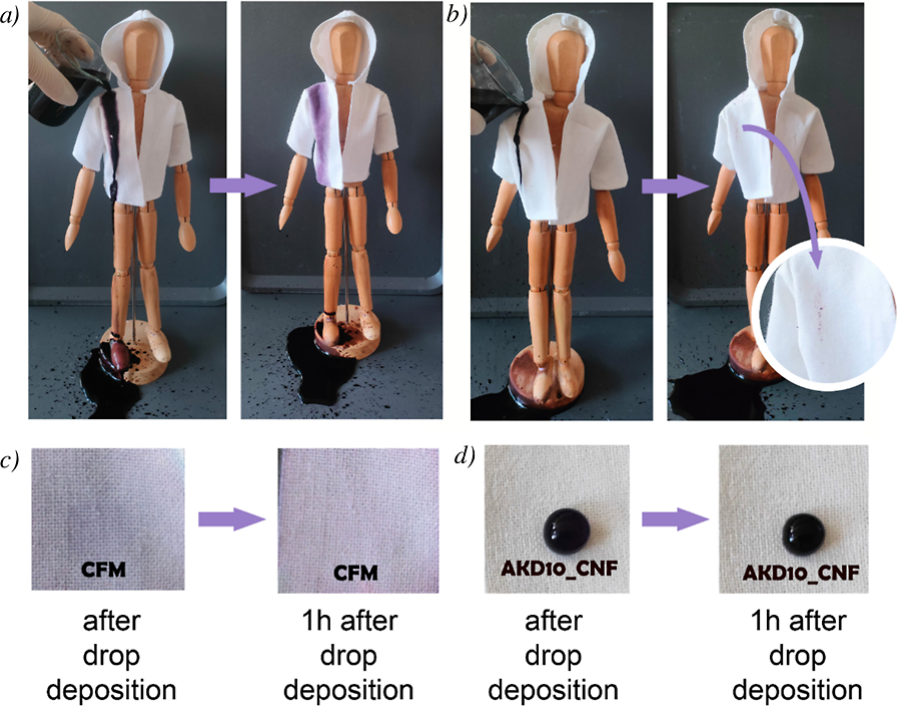
Visual water repellency tests of untreated cellulosic fiber material (CFM) and AKD– polysaccharide (AKD10_CNF) coating. (a) Water poured onto an untreated CFM garment spreads rapidly over the fabric surface. (b) Water poured onto the AKD10_CNF–coated garment is repelled and rolls off, forming discrete droplets on the surface. (c) Water droplet deposited on untreated CFM immediately after deposition and after 1 h, showing rapid absorption into the fabric. (d) Water droplet deposited on AKD10_CNF-coated CFM immediately after deposition and after 1 h, demonstrating that the droplet remains on the surface without soaking into the fabric.

#### 3.2.3 Wetting Behavior and Its Role in AKD–Cellulose Interactions

Controlling surface tension is crucial for optimizing AKD–polysaccharide coatings. Dispersions with lower surface tension wet fibers more efficiently, improving AKD spreading and penetration into the fabric structure. This enhanced wetting increases contact with cellulose hydroxyl groups, promoting the formation of β-keto ester bonds, which are responsible for durable hydrophobicity.^10,23^ The surface tension results are represented in Figures 6c and S8. The data indicate that AKD lowers the surface tension of the coating dispersion. This reduction occurs because AKD acts as a surfactant-like additive, inserting its hydrophobic alkyl chains at the liquid–air interface and disrupting water’s cohesive hydrogen-bond network.^45^ This trend is evident from the substantial drop in water’s surface tension when AKD is added at 5 or 10 wt %, while the effect at 1 wt % remains relatively modest. However, the AKD1–polysaccharide coatings still exhibited lower surface tensions, indicating that the presence of polysaccharides enhanced the liquid’s spreading ability and template role, resulting in more uniform coatings as also confirmed by the literature.^28^ This is particularly important because the hydrophobic or even superhydrophobic WCA values of the treated CFM are primarily achieved through a homogeneous distribution of AKD molecules and the directional alignment of their hydrophobic tails on the fiber surface.^23^ The AKD_CNF and AKD_agar_NH samples exhibited a correlation between lower dispersion surface tension and increased WCA. The overall uniformity of the coatings was also confirmed by WCA measurements performed at five different locations on each sample, which showed negligible deviations. Further evidence of uniform distribution was provided by SEM–EDX analysis and analysis of geometrical surface roughness. Given that the CFM is superhydrophilic, its surface energy is likely high, near the surface tension of water.^46^ All coating dispersions display lower surface tension than the estimated surface energy of CFM, thus coatings are expected to spread effectively on the CFM surface. The highest surface tension, 66 mN/m, was observed in the AKD1_CNF coating, indicating stronger intermolecular forces and reduced wetting capability. The AKD_alginate coating dispersions displayed a notable trend in surface tension, with the lowest value (48 mN/m) observed at the lowest AKD concentration. At higher AKD loadings (5 and 10 wt %), surface tension increased. This behavior can be attributed to the decreasing alginate-to-AKD ratio at higher AKD contents, which limits the availability of alginate chains to fully encapsulate each droplet. Despite this, the coated samples maintained high WCAs indicating effective wettability of the coating solution. During drying, the alginate network further directs AKD distribution on the fiber surface, as confirmed by SEM, producing a uniform coating that enhances hydrophobic performance.^47^

**Figure 6:**
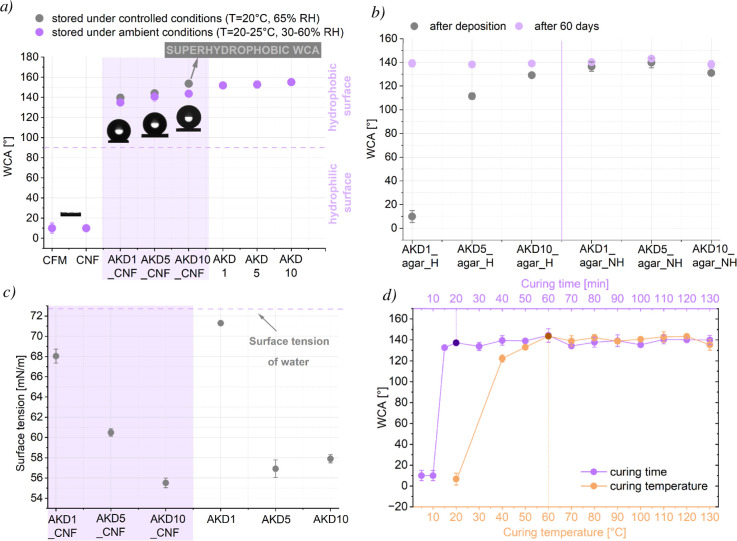
(a) WCA measurements of AKD–polysaccharide (AKD_CNF) coatings with different AKD loadings (AKD1_CNF, AKD5_CNF, and AKD10_CNF), and corresponding AKD–only reference samples, polysaccharide–only sample (CNF), and untreated cellulose fiber material (CFM), showing a pronounced increase in hydrophobicity for AKD_CNF samples with increasing AKD content and WCA values exceeding 140°. (b) Evolution of WCA over a 60 day aging period, showing a gradual increase in hydrophobicity for AKD_agar coatings prepared under heated (AKD_agar_H) and nonheated (AKD_agar_NH) conditions. (c) Surface tension of coating dispersions for AKD1_CNF, AKD5_CNF, AKD10_CNF, and reference AKD formulations, showing a decrease in surface tension with increasing AKD content and a further reduction when combined with polysaccharides. (d) Effect of curing temperature and curing time on WCA of AKD–polysaccharide coatings, indicating optimal hydrophobic performance at 60 °C and a curing time of 20 min.

Surface tension measurements indicate that polysaccharides may function as effective emulsion stabilizers and templating agents for AKD, thereby preventing AKD droplet fusion during impregnation.^28^ The mechanism varies slightly, depending on each polysaccharide and its inherent properties. Specifically, a more amorphous, plasticized, and porous polysaccharide network facilitates the diffusion, spreading, and reorientation of AKD molecules, thereby promoting the efficient formation of a hydrophobic surface. In contrast, a rigid, highly crystalline, or densely cross-linked network restricts molecular mobility.^48^

Alginate with its anionic chains most likely adsorb at the AKD oil–water interface, lowering interfacial tension and prevent droplet coalescence.^49,50^ For CNF, starch, and agar, surface tension decreased more gradually and uniformly compared to alginate. CNF forms a rigid, entangled fibrillar network that can physically stabilize AKD particles, preventing aggregation^51^ while starch’s flexible amylose/amylopectin chains offer hydrogen bonding and steric shielding that effectively prevent droplet coalescence.^52^ Agar’s gel-like double-helix network further entraps and disperses AKD droplets in a uniform manner.^53^

The surface tension results can influence practical implications. The differences in surface tension and coating uniformity can influence screen printing quality, drying kinetics, and final product performance. Polysaccharides that stabilize AKD more effectively lead to more uniform coatings, reduced drying defects, and improved hydrophobicity and mechanical integrity of the final coated product.^54,55^

### 3.3 AKD–Polysaccharide Coatings Surface Morphology and Topography

To evaluate the uniformity of coating deposition, the samples were first visually inspected (Figure S9a) for macroscopic surface irregularities. However, no anomalies were observed with the naked eye. The geometrical surface roughness was measured in order to better understand and optimize the surface properties of the coated samples, which can influence functionality, aesthetics, and comfort (fabric feel on the skin) expressed as surface mean deviation (SMD). All samples exhibited SMD (Table 1) within a relatively narrow range of 5.7 to 7.0 μm, compared to the untreated CFM, which exhibited a value of 6.1 μm. This observation indicates that the coating layer conforms closely to the inherent surface topography of the textile substrate, rather than filling in or masking its structural features. The preservation of the geometrical roughness suggests that the coating was applied in a uniform and continuous manner, without the formation of surface-level accumulations, film discontinuities, or localized thickening. This finding is further supported by the SMD plots (Figure S9b), which show that the fluctuation profiles of both coated and uncoated samples exhibit similar patterns and amplitude distributions. Additionally, the coating thickness remains within a comparable range across different areas of the sample. No statistically significant difference in roughness was observed between warp and weft directions, indicating isotropic coating behavior.

**Table 1:** Average SMD Values of AKD–polysaccharide Coatings and Untreated CFM, Calculated from Warp and Weft Measurements with Corresponding Standard Deviations, Indicating Uniform Coating Distribution and Negligible Changes in Surface Geometrical Roughness

Sample name	SMD (μm)	St. dev (warp/weft)
**AKD10_alginate**	6.02	0.326448
**AKD10_CNF**	6.25	0.152264
**AKD10_starch_H**	5.73	0.1426
**AKD10_starch_NH**	7.02	0.346482
**AKD10_agar_H**	6.60	0.379481
**AKD10_agar_NH**	6.91	0.327626
**CFM**	6.14	0.937152

As the sample morphology on the micro scale is closely linked to wettability, SEM analysis was conducted further. As shown in Figure 7, untreated CFM has a smooth surface with defined longitudinal grooves, indicative of its natural cellulose structure. AKD–polysaccharide coating surface have no visible grooves. The coating sufficiently covers the entire surface of the CFM and fills the spaces between the fibers. This is most likely due to the water-based dispersion, as water molecules are absorbed not only on the surface but also penetrate into the fabric structure along with the liquid AKD. This leads to enhanced hydrophobicity and durable fabrics.

**Figure 7:**
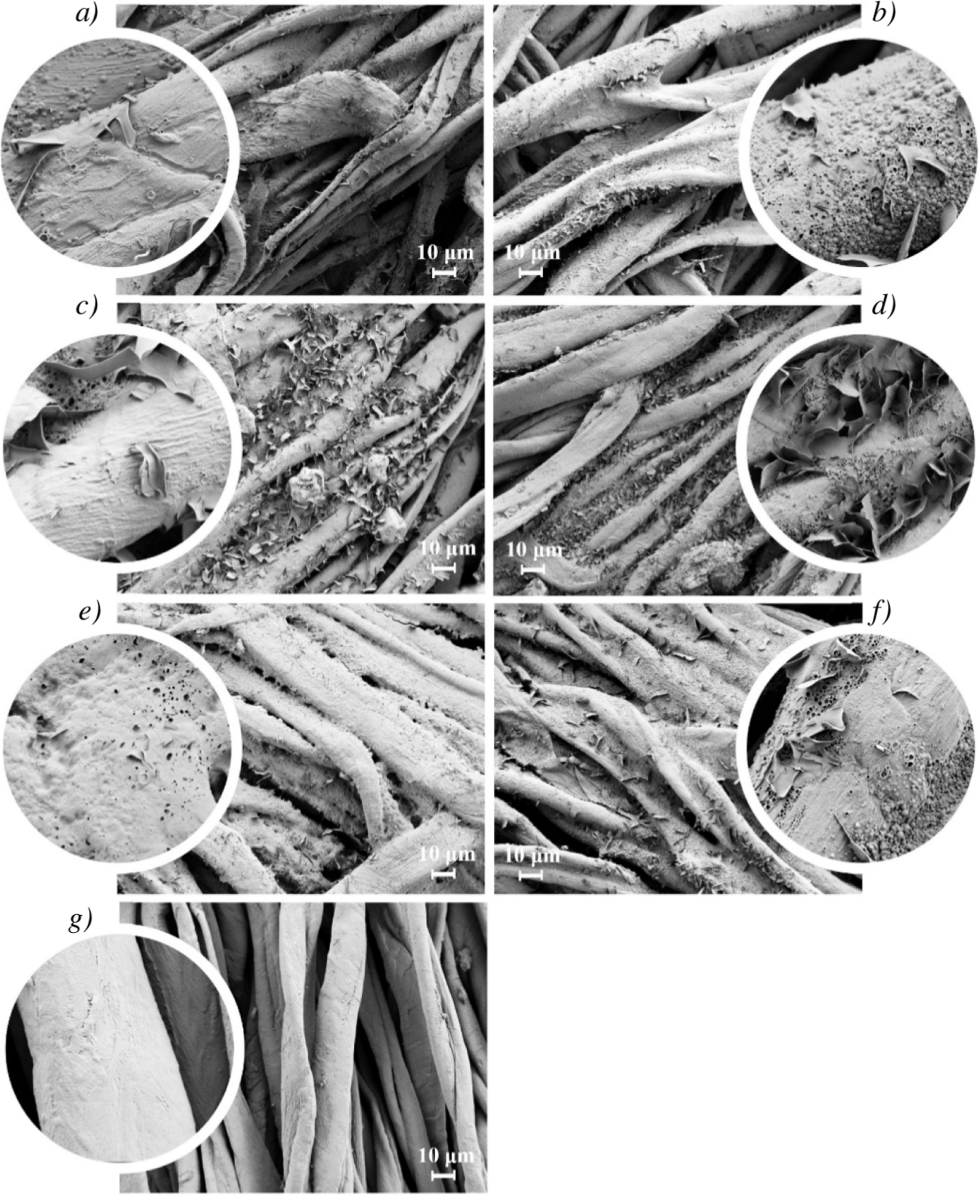
SEM images acquired at 1000× and 10 000× magnifications, showing flake-like AKD particles contributing to the microscale surface roughness of the AKD–polysaccharides coatings for: (a) AKD10_alginate, (b) AKD10_CNF, (c) AKD10_ starch_H, (d) AKD10_starch_NH, (e) AKD10_agar _H, (f) AKD10_agar_NH, while (g) untreated cellulosic fiber material (CFM), exhibits a smoother surface.

The flat, flake-like structures, which contribute to the layered appearance, are likely AKD, consistent with other studies,^23,45,56^ and concluding from the fact that AKD presence was confirmed in all samples by FTIR (Figure S1). The flakes mostly range from 1 to 3 μm, which confirms micro surface roughness of coated samples, which can increase water repellency via the lotus-like leave effect.

SEM analysis highlights two distinct samples: AKD_starch_H and AKD_agar_H. In the AKD_starch_H coating, large granules (>10 μm) are visible, which can be attributed to starch aggregates formed during heating. Smaller and less frequent granular features of varying morphology are also observed across other samples; these likely represent polysaccharide domains that did not fully mix with AKD and consequently aggregated into microstructures. Such granule formation may also result from heat-induced crystallization of polysaccharides.

In contrast, the AKD_agar_H sample shows noticeably reduced surface roughness, indicating that AKD is predominantly positioned beneath the agar layer. This subsurface distribution is consistent with FTIR results and helps explain the lower water contact angle of this sample. The heated agar network may restrict AKD mobility and prevent proper surface spreading or orientation of its hydrophobic chains. The resulting smoother surface—combined with limited AKD exposure—further diminishes hydrophobicity, as surface roughness is known to enhance water repellency.^57^

### 3.4 AKD–Polysaccharide Coatings’ Physical Properties

In the following step, we also examined the physical properties of the AKD–polysaccharide coatings on CFM, as these parameters are crucial for their practical use and consumer acceptance, particularly in terms of comfort, durability, and overall material performance. Gravimetric analysis revealed that the coating accounts for 3 ± 2% of the total mass of the treated samples, indicating a lightweight coating layer. The thickness of the coatings was calculated based on the thickness of the coated samples compared to uncoated samples, ranging around 0.04 mm (±0.03 mm). While the thickness of the coating can potentially influence the mechanical properties of the samples, the differences in this case were too small to have a noticeable impact. Mechanical properties presented in Figures 8a,b and S10, S11, specifically tensile strength and elongation, were measured to assess whether the coating altered the properties of the CFM. The measurements were taken in both the warp and weft directions. The untreated CFM exhibited tensile strengths of 544.83 N (warp) and 540.11 N (weft), with elongations of 17.85% and 23.53%, respectively. Mechanical testing showed that both tensile strength and elongation were primarily influenced by fiber-coating interactions (as confirmed by FTIR and further supported by SEM observations), as also reported elsewhere.^58^ Elongation measurements showed a more pronounced trend toward increased flexibility, particularly in starch-containing coatings. The AKD_starch samples exhibited the largest increases in elongation: AKD1_starch_NH_warp increased by 6.42%, while the AKD10_starch_NH_weft and AKD5_starch_NH_weft samples increased by 6.21% and 6.34%, respectively. This behavior is consistent with the known plasticizing effect of starch, which introduces flexibility into the coating matrix and permits limited fiber mobility, especially when the film retains residual moisture or is not strongly cross-linked. In contrast, coatings with higher AKD concentrations tend to be more rigid, as AKD forms crystalline or wax-like domains that restrict fiber movement^59^ and therefore reduce elongation. As a result, lower AKD concentrations generally enable greater elongation relative to untreated CFM, whereas higher concentrations either reduce elongation or maintain it near the values of the untreated CFM. Among the polysaccharides used, starch is the only one capable of acting as a plasticizer,^29^ which explains why elongation increases were largely confined to starch-based coatings despite the general stiffening effect of AKD. Overall, the observed variations in elongation reflect the balance between the flexibility contributed by the polysaccharide matrix and the rigidity introduced by AKD.

**Figure 8:**
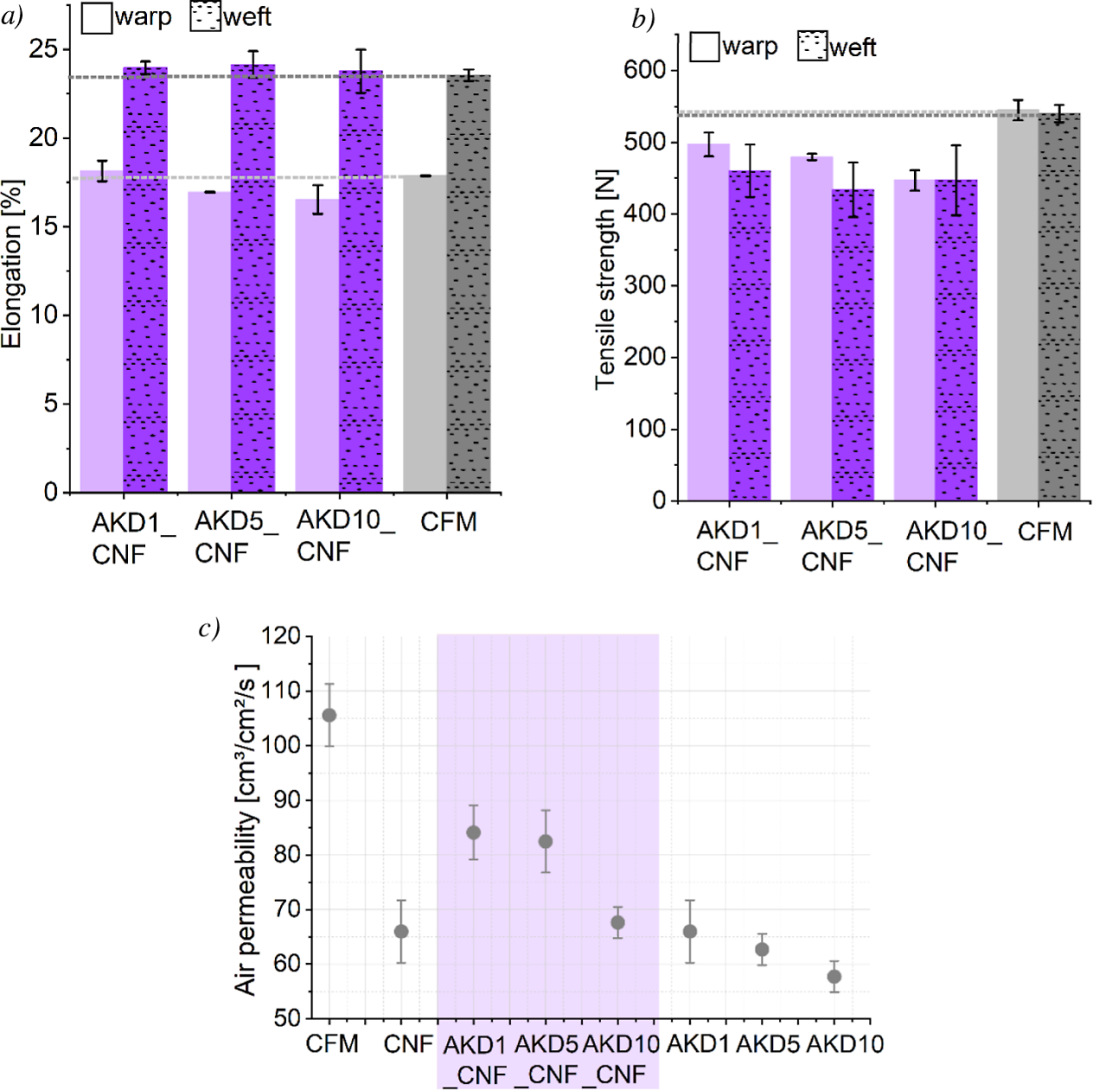
(a) Results of elongation at break of untreated cellulosic fiber material (CFM) and AKD–polysaccharide coatings (AKD_CNF) show that elongation decreases for AKD5_CNF and AKD10_CNF, while an increase is observed for AKD1_CNF in warp direction, whereas in the weft direction, elongation increases for all AKD_CNF samples. (b) Results for tensile strength show a decrease for AKD_CNF samples compared to untreated CFM. (c) Air permeability of AKD_CNF samples and reference samples­(CFM, polysaccharide–only (CNF), and AKD–only samples) shows that all coating components reduce air permeability, with lower AKD loadings associated with higher air-permeability.

Most AKD–polysaccharide coatings reduced tensile strength in both directions. The largest reduction was observed for the AKD_starch_NH_weft sample (−126 N), while AKD5_agar_H_warp showed the smallest decrease (−9 N). The only formulation that increased tensile strength was AKD1_agar_H, which showed gains of 15 N (warp) and 18 N (weft). The reduction of tensile strength can be attributed to several interacting effects. The applied AKD-polysaccharide coating interactions restricts the natural mobility of the cotton fibers, limiting their ability to slide and realign during deformation.^59,60^ This reduced flexibility compromises the fabric’s capacity to redistribute stress, leading to lower tensile strength and elongation. In addition, the hydrophobic AKD component partially masks cellulose hydroxyl groups, diminishing the interfiber hydrogen bonding that normally contributes to efficient stress transfer within the fiber bundle. Higher AKD concentrations intensify this effect, as the formation of rigid, crystalline, or wax-like AKD domains further disrupts interfiber interactions and stiffens the coated fibers.^58^ The polysaccharide matrix also contributes to the mechanical response: starch and agar tend to form relatively brittle or crystalline films, particularly after heating, which can introduce internal stresses or microstructural discontinuities that weaken the fiber–coating interface.^43,61^ In contrast, more flexible matrices such as alginate or CNF impart less brittleness but still limit fiber motion.^43,62^ The only exception, AKD1_agar_H sample, likely gains strength due to the dominant presence of agar, which forms a cohesive and uniform film capable of enhancing adhesion between fibers without excessively restricting their movement.^63^ Although heating at 60 °C can promote limited AKD–cellulose bonding, these potential strengthening effects appear overshadowed by the mobility restrictions and coating-induced brittleness that collectively reduce mechanical performance in the majority of formulations. These findings highlight the importance of carefully optimizing AKD/polysaccharide ratios to achieve desirable hydrophobicity without compromising the mechanical integrity of cellulosic fibers.

Air permeability values (Figures 8c and S12) are also important as maintaining adequate porosity ensures the fabric remains breathable while achieving effective hydrophobic treatment. AKD–only samples show a clear trend of decreasing breathability compared to untreated CFM (105.58 cm^3^/cm^2^/s) with increasing AKD concentration. The AKD1 coating exhibits an air permeability of 61 cm^3^/cm^2^/s, while higher AKD loadings (5% and 10%) expectedly further reduce permeability to values around 55 and 50 cm^3^/cm^2^/s, respectively. AKD forms a compact, hydrophobic polymer layer that effectively seals the pores, preventing air passage.^64^ Similarly, was observed in a study by Yang et al.,^65^ where the air oxygen permeability decreased significantly with higher concentration of AKD. When polysaccharides are applied alone (without AKD), alginate shows the highest air permeability (∼85 cm^3^/cm^2^/s), followed by CNF and agar_NH, which also maintain relatively high values. This suggests that these polysaccharide layers form open and breathable networks when deposited individually. The addition of AKD to polysaccharides generally reduces air permeability, reflecting the Yang et al.,^65^ where the air oxygen permeability decreased significantly with higher concentration of AKD. When polysaccharides are applied alone (without AKD), alginate shows the highest air permeability (∼85 cm^3^/cm^2^/s), followed by CNF and agar_NH, which also maintain relatively high values. This suggests that these polysaccharide layers form open and breathable networks when deposited individually. The addition of AKD to polysaccharides generally reduces air permeability, reflecting the densifying and film-forming nature of AKD. As observed in the SEM images, despite a thin and lightweight coating layer, the coating partially or completely closes the pores between cellulosic fibers. A notable exception is observed with alginate at 1% AKD, where the air permeability (∼83 cm^3^/cm^2^/s) remains almost identical to that of pure alginate, suggesting that at low AKD concentration, the coating structure remains open and minimally affected. However, as the AKD concentration increases to 5% and 10%, the permeability of the alginate-based coatings drops significantly, mirroring the trend seen in other combinations. Moreover, heat treatment further reduced air permeability, particularly in AKD_starch_H samples, which exhibited the lowest permeability values among all coatings, although still above 50 cm^3^/cm^2^/s. The reduced air permeability in heat-treated starch-based systems may result from thermal-induced changes in starch morphology, such as loss of crystallinity and short-range molecular order, as previously reported by Reyes et al.^66^ These transformations likely lead to a denser, less porous coating, hindering airflow.

### 3.5 Coating Stability

To evaluate the suitability of coated samples for practical applications, treated cellulosic material samples were subjected to stability testing under simulated conditions. Chemical stability test involved exposure to acidic, neutral and alkaline environments for 1, 24, and 48 h (Figures 9a, S13–S15). AKD–polysaccharide coatings exhibited strong stability across all pH levels, retaining hydrophobicity even after 48 h, with 1–10% WCA decrease for most samples. This behavior was more pronounced at higher AKD concentrations, as confirmed by FTIR analysis, which showed sustained intensity of C–H stretching vibrations despite elevated – OH peaks, indicating the preservation of hydrophobic functionality. In contrast, AKD–only samples maintained stability in neutral and alkaline media but showed significant degradation in acidic environments, particularly at lower concentrations (1 and 5 wt %). This instability could be attributed to acid-catalyzed hydrolysis of the ketene ring, forming β-keto acids that decarboxylate into unreactive ketones, thereby reducing ester bond formation with cellulose and compromising hydrophobic performance, as previously described by Lindström and Kumar et al.^10,36^ The superior acid resistance of AKD–polysaccharide coatings (except for AKD1_agar_H and AKD5_agar_H) is likely due to polysaccharides working as functional stabilizers, improving the chemical resistance of the coating matrix. Polysaccharides may also act as hydrophilic physical barriers,^67,68^ that can restrict proton penetration and moderate AKD release, facilitating controlled esterification with cellulose, as also described by Liu et al.^28^ FTIR spectra supported this mechanism, with stable β-keto ester-associated peaks that shifted at 1850 cm^–1^ and 1723 cm^–1^ across different pH conditions. In our study, these peaks appeared even in samples where they were initially barely detectable. These signals were most strongly observed in the AKD_alginate sample (Figure 9b), highlighting alginate’s effectiveness in enhancing chemical stability. The rapid loss of hydrophobicity in AKD1.5_agar_H after just 1 h, underscores the critical role of interfacial stabilization^28^ in coating longevity. Gravimetric analysis further confirmed coating retention, with a maximum weight change of only 0.009 g, indicating minimal material loss and strong surface adhesion across all test conditions.

**Figure 9:**
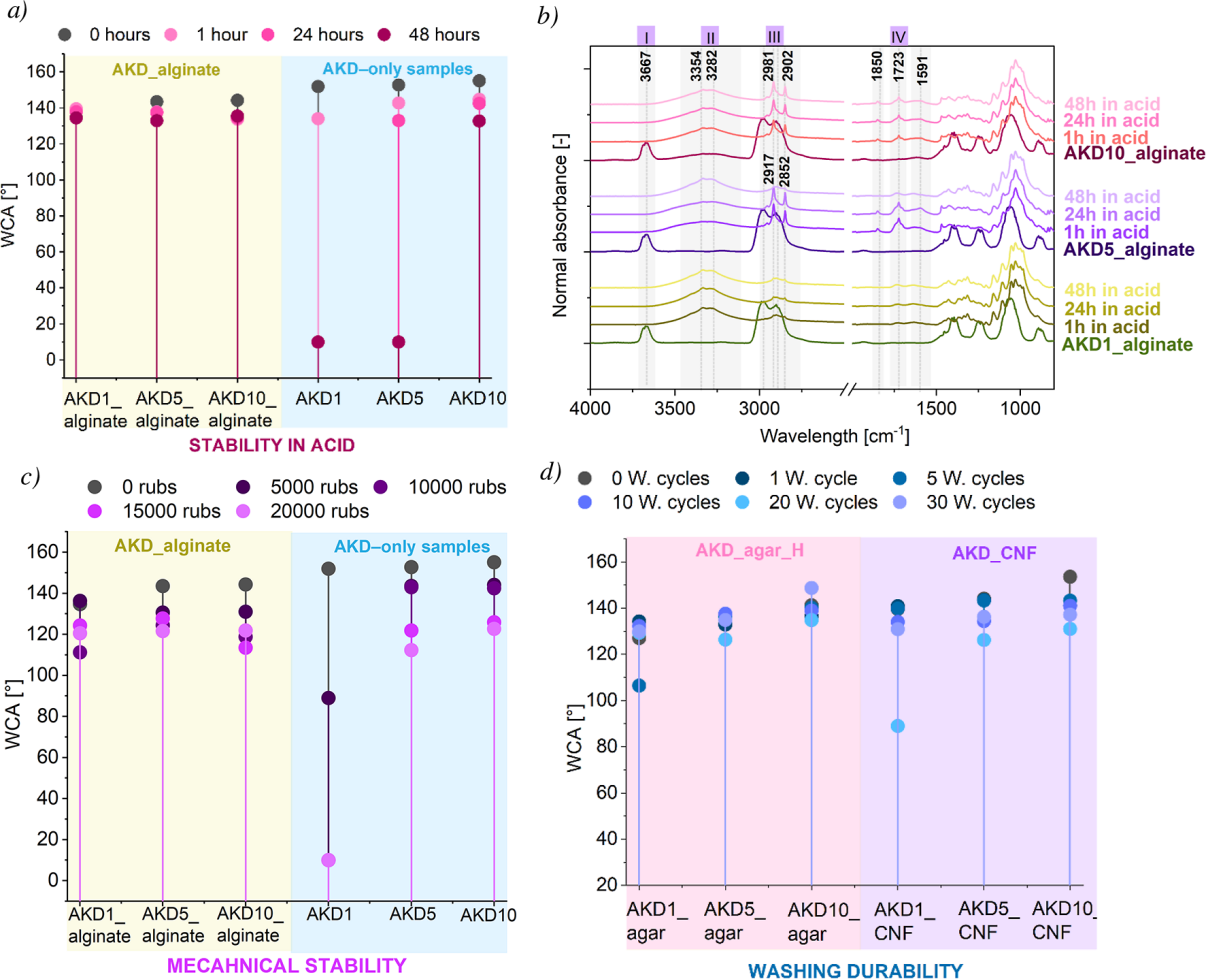
Stability of AKD–polysaccharide coatings. (a) Acid stability, showing higher resistance of AKD–polysaccharide coatings compared to AKD–only coatings after 48 h exposure. (b) FTIR spectra of the AKD_alginate coating after acid exposure, indicating a decrease in CH stretching bands and an increase in OH-related bands. (c) Mechanical stability (rubbing resistance), demonstrating superior performance of the AKD_alginate coating relative to AKD–only coatings. (d) Washing stability of AKD_alginate and AKD_CNF coatings, showing improved washing resistance at higher AKD concentrations.

The mechanical stability of the developed coatings was further evaluated through standardized abrasion testing, as presented in Figures 9c and S16a. Coatings composed of AKD in combination with polysaccharides—particularly alginate and starch_NH—demonstrated better abrasion resistance compared to AKD_only coatings. This mechanical degradation is consistent with previous reports, where abrasion was shown to disrupt the ordered alkyl layer of AKD, resulting in a reduction in surface hydrophobicity.^10,36^ Our findings confirm that most polysaccharides contribute positively to the abrasion stability at 1 wt % AKD, likely by supporting film integrity and anchoring the AKD network. AKD–polysaccharide samples with a 10 wt % AKD content exhibited superior retention of hydrophobicity under mechanical stress, maintaining high WCA even after 20 000 cycles. Among all durability tests, AKD–polysaccharide coatings showed the lowest resistance to abrasion, with most samples exhibiting a 10–35% WCA reduction. FTIR analysis (Figure S17) further confirmed abrasion-induced chemical changes, including decreased C–H stretching peaks, increased O–H peaks, and pronounced alterations in the fingerprint region—also visible in untreated cellulose—indicating that the substrate likely fractured under mechanical stress which potentially also decreased the hydrophobic performance. SEM images Figure S16b show fractures on the fibers; however, they also revealed that some AKD fragments remained, especially between fibers, which likely explains why the samples retained hydrophobicity.

Washing stability results (Figures 9d and S18a), especially for samples with 5 and 10 wt % AKD in combination with the polysaccharides, show exceptional hydrophobic stability even after 30 washing cycles, resulting in high WCA values (typically >130°, around 10% reduction). Interestingly, certain samples exhibited increased hydrophobicity after washing, with WCA surpassing those of their unwashed counterparts. This suggests that washing may induce surface restructuring or selectively remove hydrophilic components,^28^ resulting in a more hydrophobic surface. This effect is clearly visible in the SEM images (Figure S18b) of the AKD10_agar_H sample, where the agar film is no longer present and AKD domains are more prominently exposed than before, increasing initial WCA for 10%. This phenomenon is consistent with observations reported by Cao^69^ where hydrophobicity improved after acid treatment, indicating that chemical and physical surface modifications can synergistically enhance AKD-based coating. After 30 washing cycles, AKD1_polysaccharides samples generally exhibited a significant decrease in WCA, in some cases approaching hydrophilic values. This loss of hydrophobicity can be attributed to multiple degradation mechanisms: partial hydrolysis or leaching of the AKD component. Samples with higher initial AKD concentrations maintained—or even increased—their WCA despite undergoing more washing cycles. Although the FTIR spectra (Figure S19) after 30 cycles show reduced C–H stretching intensity compared to 20 cycles, indicating partial AKD loss, the simultaneous decrease in O–H absorption suggests that hydrophilic surface groups were also removed or masked. This reduction of accessible O–H groups can lower overall surface polarity^70^ and increase apparent hydrophobicity, even when some AKD is lost. Additionally, repeated washing may reorganize the remaining AKD domains, promoting reorientation of hydrophobic alkyl chains toward the surface.

## 4 Contextualizing This Study in the Scientific Landscape

The graphical comparison, which positions our study among previously reported AKD–only and AKD–polysaccharide coatings on cellulosic substrates, is shown in Figure 10. Additional details on the studies included in this comparison are provided in Table S1.

**Figure 10:**
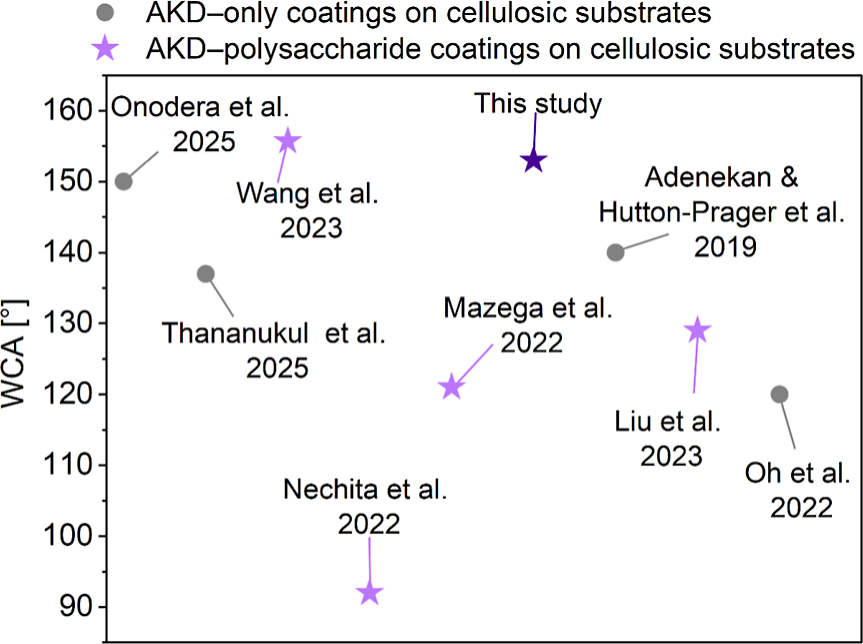
Placement of this study within the context of existing literature on AKD–only and AKD–polysaccharide coatings for cellulosic fiber materials, based on achieved WCA values, shows that the samples reported here are among the most effective.

The development of superhydrophobic cellulosic surfaces has long been dominated by purely synthetic AKD-based coatings, which routinely achieve WCA above 150°.^22,44^ However, despite their high hydrophobicity, these systems often suffer from limited biodegradable synthetic additives. Moreover, durability is frequently underreported or insufficiently evaluated in many of these studies.

In response to these challenges, recent research has increasingly explored AKD–polysaccharide hybrid coatings as more sustainable alternatives. Yet most of these hybrid systems have attained only moderate hydrophobicity (WCA 90–130°),^28,29,71^ typically due to incomplete surface coverage and the absence of well-developed hierarchical roughness.^21,71^ This persistent performance gap between AKD–only and AKD–polysaccharide formulations has remained a central limitation in the field.

The present study bridges this gap by demonstrating that a rationally engineered polysaccharide network architecture can effectively template AKD deposition, enabling the formation of superhydrophobic surfaces with a WCA of 153° in the AKD–CNF system. This performance matches leading AKD–only coatings while simultaneously offering enhanced durability and preserved biodegradability. To our knowledge, only one prior work has achieved comparable hydrophobicity within such hybrid system: Wang et al.^21^ used chitosan in combination with AKD; however, in this study, we employed different polysaccharides.

Furthermore, only two previous studies^22,23^ have investigated such hybrid coatings on textiles; the remaining work has focused predominantly on paper substrates. Textile applications introduce additional requirements—such as flexibility, air permeability, and sensory comfort—which make the translation of coating technologies particularly challenging. For the first time, the present study employs alginate and agar in combination with AKD for hydrophobization of textiles. By uniting high hydrophobicity, sustainability, durability and textile compatibility, this work establishes a new benchmark in the design of eco-friendly hydrophobic coatings for natural fibers.

## 5 Conclusions

This work presents a sustainable and durable strategy for hydrophobizing cellulose fabrics using AKD in combination with different polysaccharides. AKD efficiently imparts hydrophobicity through physical and chemical modifications, including increased microscale roughness and covalent β-keto ester bond formation with cellulose, enhancing coating durability. Polysaccharides remain physically incorporated, stabilizing the AKD emulsion, preventing droplet fusion, and ensuring uniform AKD distribution, as evidenced by surface tension measurements and SEM. Their intrinsic properties—hydroxyl density, accessibility, porosity, chain mobility, crystallinity, and hydrophilic–hydrophobic balance—significantly influence coating formation and performance.

The resulting thin coatings exhibited high and durable hydrophobicity, in some cases approaching superhydrophobic behavior with roll-off-like effects. In general, stability tests showed minimal reductions in WCA, decreasing less than 10% after laundering, 1–10% after chemical stability tests, and 10–35% after abrasion, confirming their suitability for practical textile applications. Mechanical and comfort-related properties were moderately affected by coating composition: tensile strength typically decreased by 1–25%, while elongation ranged from slight reductions to increases of up to 36%. Air permeability decreased by 20–50%. Each polysaccharide conferred distinct advantages in hydrophobicity, durability, mechanical performance, and breathability, indicating that future hybrid polysaccharide systems could further optimize overall coating performance.

In summary, the AKD–polysaccharide system offers a biobased, water-processable alternative to conventional PFAS- and silane-based hydrophobic treatments. By combining durability, functionality, and favorable tactile properties with renewable, water-based processing, this approach advances environmentally responsible surface treatments. Beyond textiles, these materials hold significant potential for applications in cosmetics, food packaging, and other areas requiring safe, multifunctional, and sustainable surface protection.

## Supplementary Material



## Notes

Supplementary results: FTIR spectra for all samples, EDX results for all samples, results of sequential hexane extraction for AKD10_CNF sample, FTIR spectra of AKD10_CNF at different curing temperatures and times, WCA result of all samples, water absorption behavior for all samples, surface tension measurements for all samples, scan images of CFM and AKD_agar_NH sample, SMD plots of CFM and AKD10_CNF sample, elongation, tensile strength, air permeability results for all samples, WCA after stability tests for all samples, FTIR spectra after mechanical stability test for all samples, FTIR spectra after washing stability test for AKD_CNF samples, SEM images of the AKD10_CNF after mechanical stability test and AKD10_agar_H sample after washing stability test, summary of reported AKD–only and AKD–polysaccharide coatings on cellulosic substrates (PDF)

## Abbreviations

AKD: alkyl ketene dimer
ATR-FTIR: attenuated total reflectance- Fourier transform infrared spectroscopy
CFM: cellulosic fiber material
CNF: cellulose nano fibers
EDX: energy dispersive X-ray spectroscopy
PFAS: per- and polyfluoroalkyl substances
SEM: scanning electron microscopy
XPS: energy-dispersive X-ray spectroscopy
UV: ultraviolet
WCA: water contact angle
ZP: zeta potential
