# Supplementary material for: Sustainable, Safe, and Effective (Super)Hydrophobic Coatings for Cellulosic Fiber Material via Alkyl Ketene Dimer and Polysaccharide Integration
Source: ACS Sustain Chem Eng. 2026 Feb 23;14(9):4329–46. doi: 10.1021/acssuschemeng.5c08955 (PMC12977283; doi:10.1021/acssuschemeng.5c08955)
Supplement: Supplementary file 1 [file sc5c08955_si_001.pdf]

Supporting Information

Sustainable, Safe, and Effective (Super)Hydrophobic Coatings for Cellulosic Fiber Material via Alkyl Ketene Dimer and Polysaccharide Integration

Petra Jerič[a,b], Barbara Golja[c,d], Gregor Lavrič[d,e], Janvit Teržan[d], Anja Verbič[d*], Blaž Likozar[d], Uroš Novak[d*]

[a] Jožef Stefan International Postgraduate School, Jamova 39, 1000 Ljubljana, Slovenia

[b] Faculty of Mechanical Engineering, University of Maribor, Smetanova ulica 17, 2000 Maribor, Slovenia

[c] Faculty of Natural Sciences and Engineering, University of Ljubljana, Snežniška ulica 5, 1000 Ljubljana, Slovenia

[d] Department of Catalysis and Chemical Reaction Engineering, National Institute of Chemistry, Hajdrihova 19, 1000 Ljubljana, Slovenia

[e] Pulp and Paper Institute, Bogišićeva 8, 1000 Ljubljana, Slovenia

*Co-corresponding Authors: A. Verbič, U. Novak

anja.verbic@ki.si; uros.novak@ki.si

Number of pages: 23

Number of figures: 19

Number of tables: 1

Table of Contents

Figure S1. FTIR spectra of untreated cellulosic fiber material (CFM), pure AKD, reference coating–only samples (COS), and AKD–polysaccharide samples……………………………S4

Figure S2. EDX analysis showing the elemental composition (wt %) of untreated cellulosic fiber material (CFM) and AKD–polysaccharide samples…………………………………….S5

Figure S3. Results of sequential hexane extraction (4 and 8 h) for the AKD10_CNF sample. a) WCA measurements. b) FTIR spectra……………………………………………………..S6

Figure S4. FTIR spectra of AKD10_CNF sample cured at different temperatures…………..S7

Figure S5. FTIR spectra of AKD10_CNF sample at different curing times…………………S7

Figure S6. a) WCA measurements of AKD–polysaccharide coatings stored under ambient and controlled conditions. b) Photo of a water drop on the AKD10_CNF coating. c) WCA of reference polysaccharide–only, AKD–only, and CFM sample………………………………S8

Figure S7. (a) Water absorption behavior immediately after water deposition. (b) Water absorption behavior after 1h…………………………………………………………………S9

Figure S8. Surface tension measurements of the coating dispersions………………………S10

Figure S9. (a) Surface scan images of AKD10_starch_NH sample. (b) SMD plots of the CFM and AKD10_CNF sample…………………………………………………………………...S11

Figure S10. Elongation results of AKD–polysaccharide samples and reference CFM……..S12

Figure S11. Tensile strength results of AKD–polysaccharide samples and reference CFM……………………………………………………………………………………………S12

Figure S12. Air permeability results of AKD–polysaccharide coatings, reference polysaccharide–only, and AKD–only samples, and CFM…………………………………...S13

Figure S13. WCA of AKD–polysaccharide and AKD–only samples after exposure to neutral conditions………………………………………………………………………………………S14

Figure S14. WCA of AKD–polysaccharide and AKD–only samples after exposure to alkali conditions………………………………………………………………………………………S14

Figure S15. WCA of AKD–polysaccharide and AKD–only samples after exposure to acid conditions……………………………………………………………………………S15

Figure S16. (a) WCA after 20 000 rubbing cycles for AKD–polysaccharide coating and AKD–only samples. (b) SEM images of the AKD10_CNF sample after 20 000 rubbing cycles…....S16

Figure S17. Comparison of FTIR spectra of CFM and AKD10_polysaccharide samples before and after 20 000 rubbing cycles…………………………………………………………...S17

Figure S18. (a) WCA of AKD–polysaccharide samples after washing tests. (b) SEM images of the AKD10_agar_H sample before and after 30 washing cycles……………………………S18

Figure S19. FTIR spectra of the AKD_CNF samples after 10, 20, and 30 washing cycles……………………………………………………………………………..…S19

Table S1. Summary of reported AKD–only and AKD–polysaccharide coatings………S20-S22

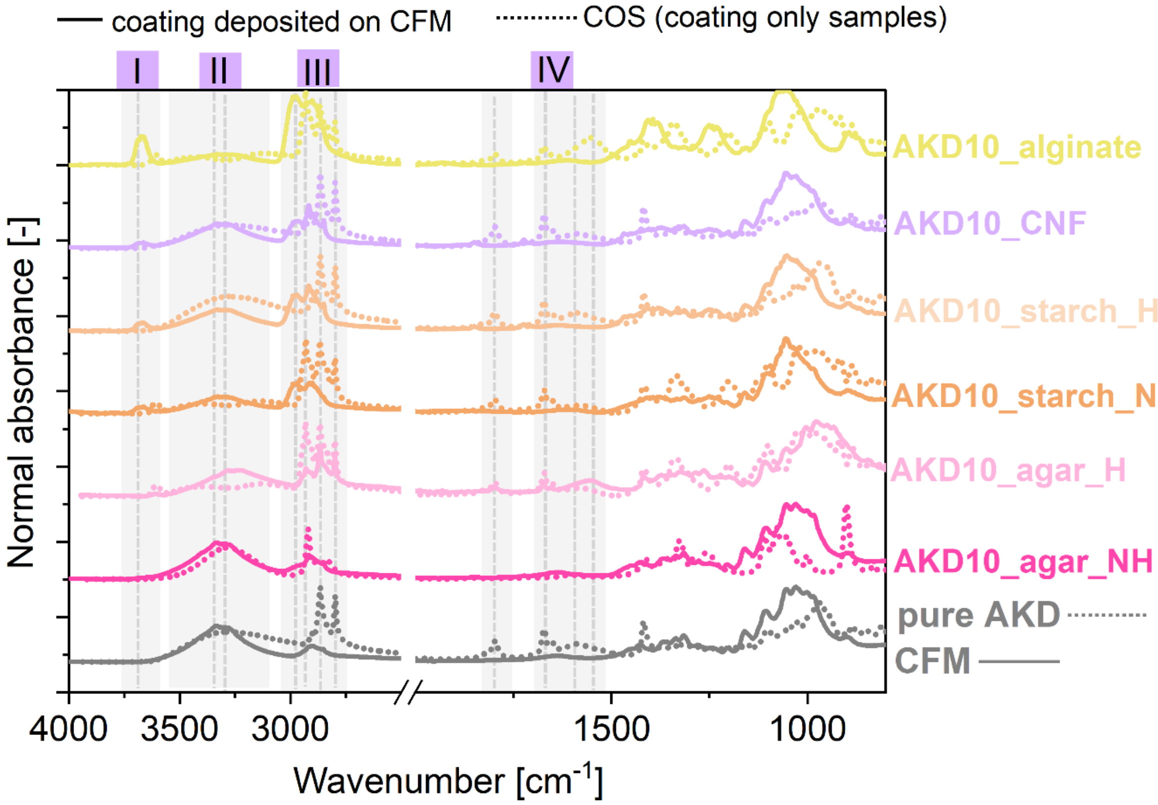

Figure S1. FTIR spectra of untreated cellulosic fiber material (CFM), pure AKD, reference coating–only samples (COS), and AKD–polysaccharide samples. Compared with untreated CFM, the AKD–polysaccharide samples exhibit increased intensity of nonpolar C–H stretching vibrations, decreased O–H intensity, and peak shifts relative to the corresponding reference COS samples, indicating successful deposition of AKD–polysaccharide coatings.

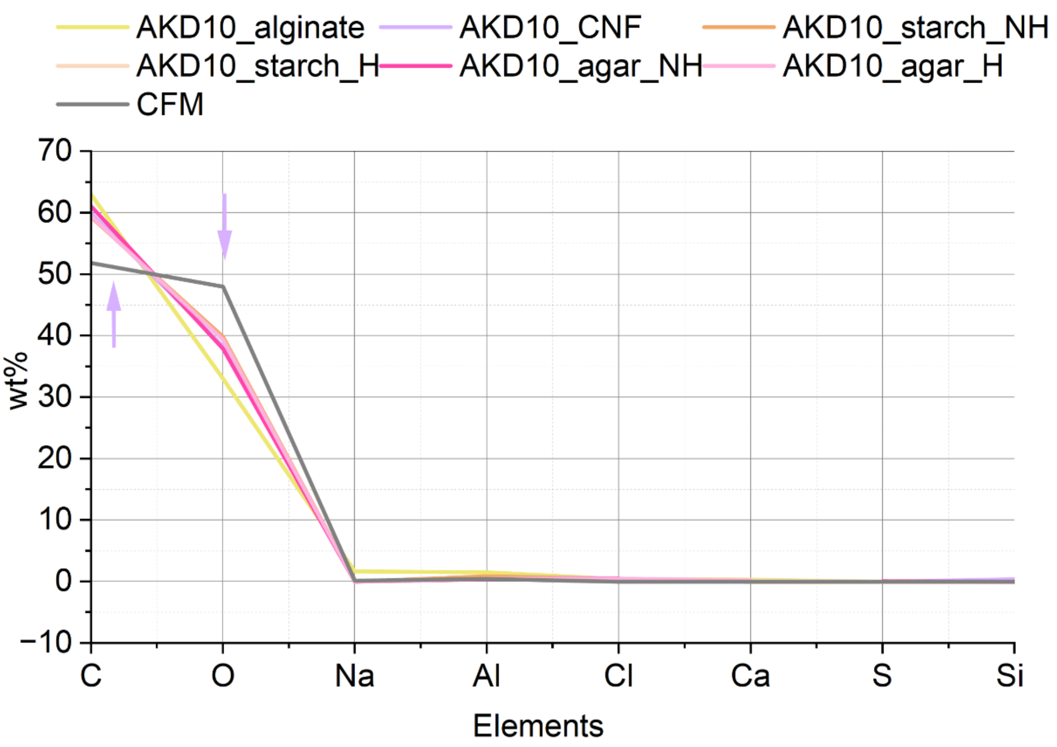

Figure S2. EDX analysis showing the elemental composition (wt %) of untreated cellulosic fiber material (CFM) and AKD–polysaccharide samples. Compared to untreated CFM, the AKD–polysaccharide coatings exhibit an increased carbon (C) content and a decreased oxygen (O) content, confirming successful surface modification.

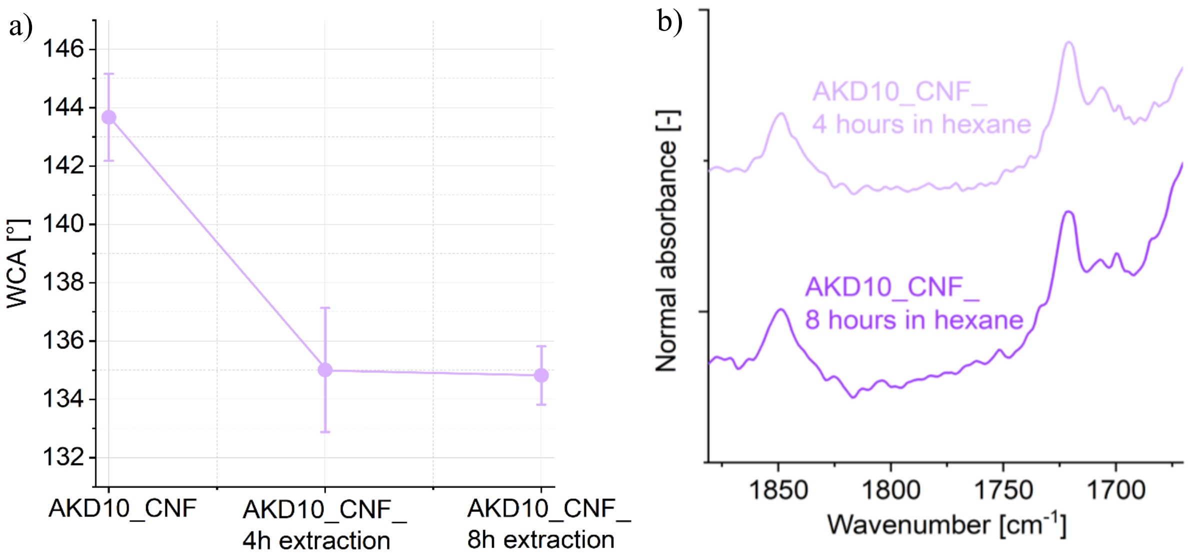

Figure S3. Results of sequential hexane extraction (4 and 8 h) for the AKD10_CNF sample. (a) WCA measurements show a slight decrease after 4 h of extraction, while not decreasing further after 8 h. (b) FTIR spectra of the extracted AKD10_CNF samples still exhibit characteristic β-keto ester peaks even after 8h of extraction, indicating that a chemical reaction occurred between AKD in the coating and the CFM surface.

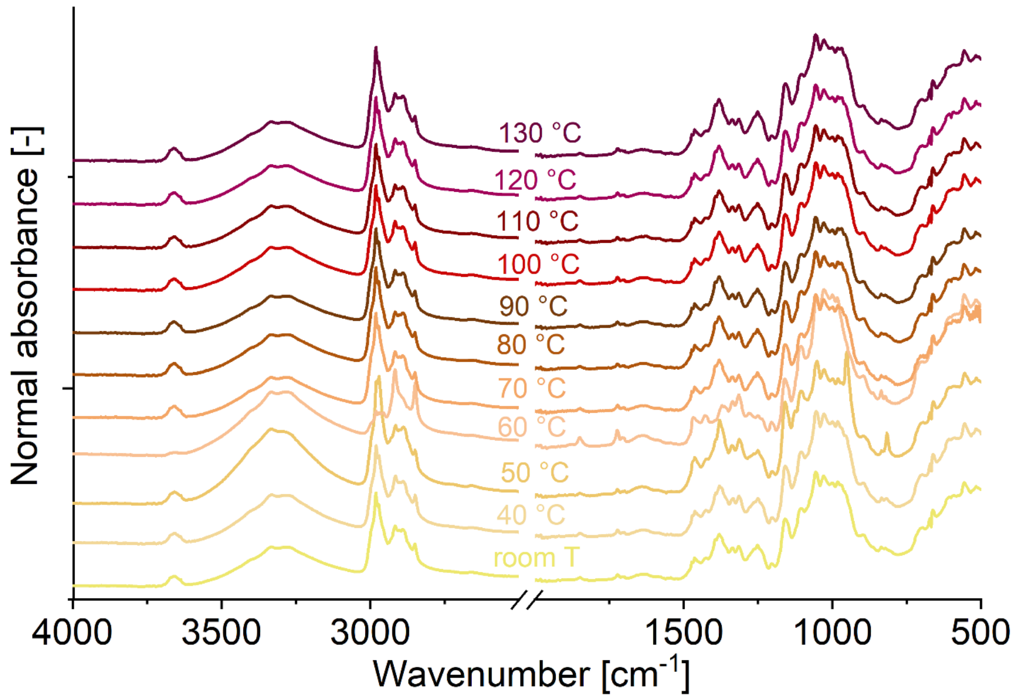

Figure S4. FTIR spectra of AKD10_CNF samples cured at different temperatures, showing similar composition and highlighting the dominant influence of uniform AKD distribution over molecular orientation.

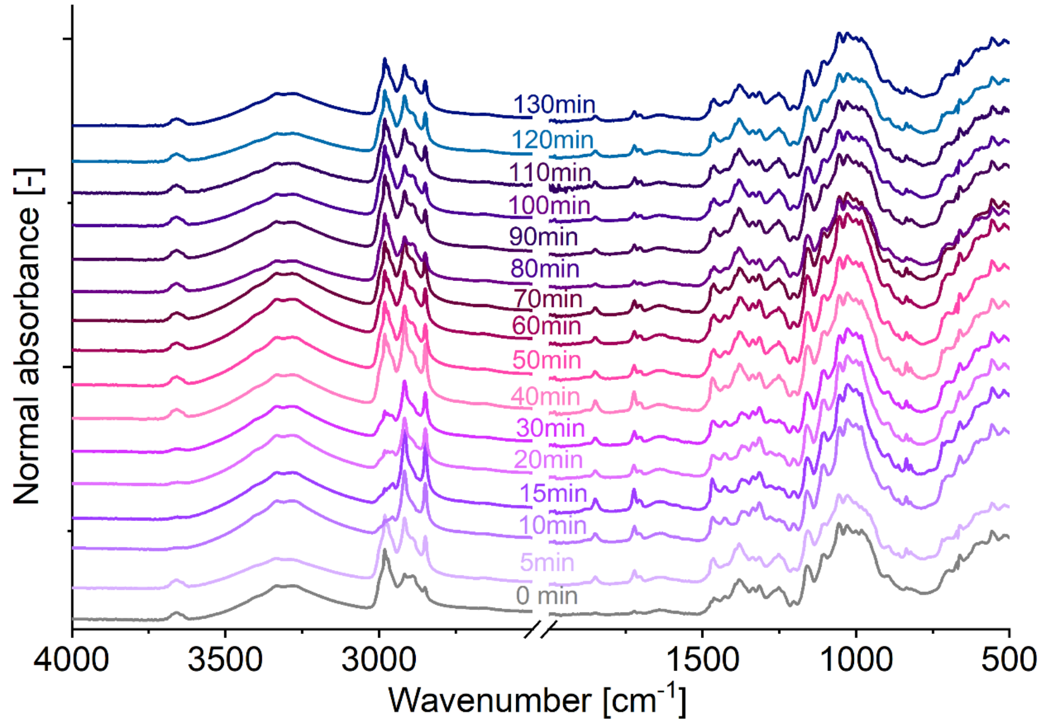

Figure S5. FTIR spectra of AKD10_CNF at different curing times, showing similar composition and highlighting the dominant role of uniform AKD distribution over molecular orientation.

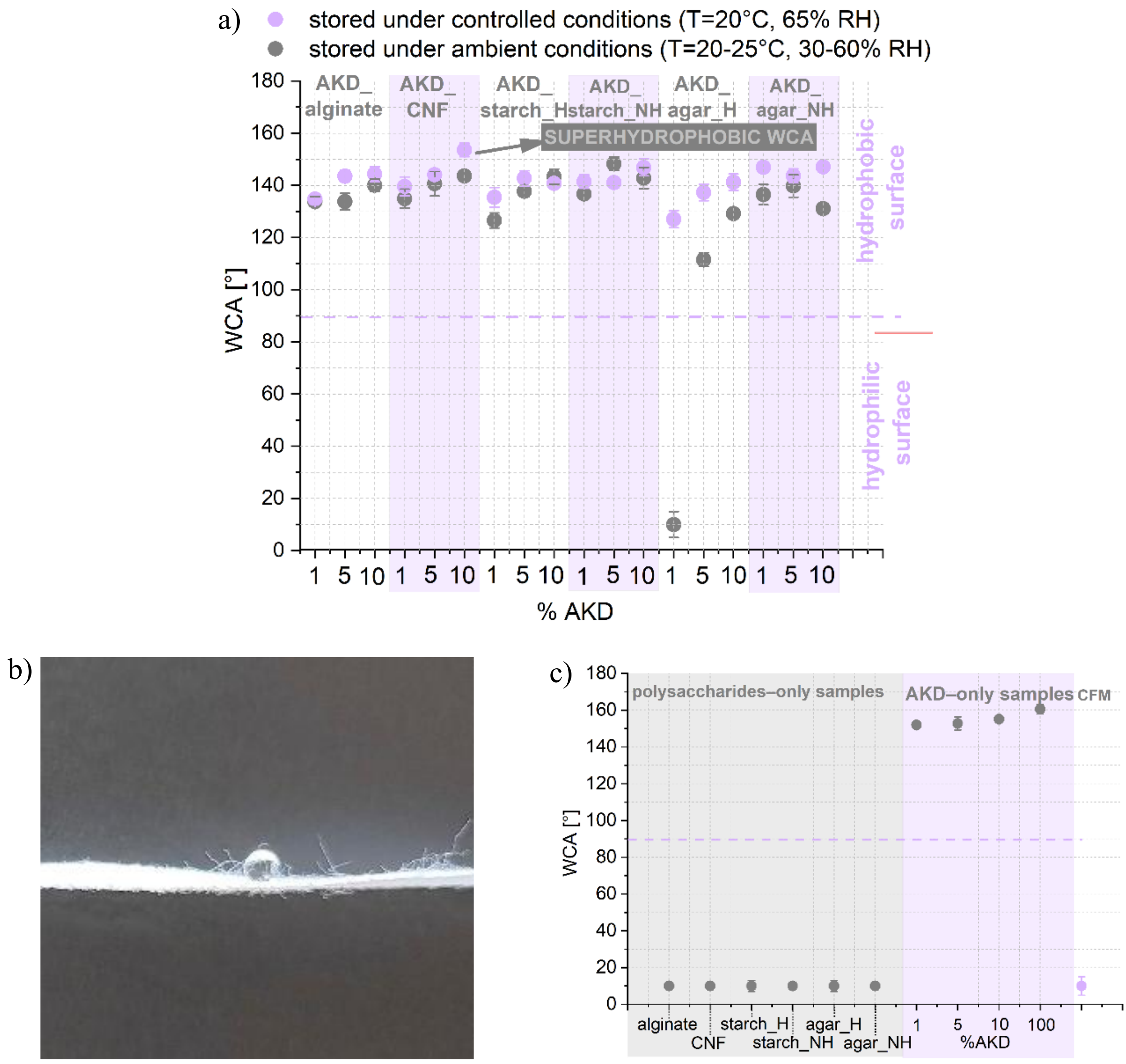

Figure S6. a) WCA measurements of AKD–polysaccharide coatings stored under ambient and controlled conditions. The coatings impart hydrophobicity to cellulosic fiber material (CFM), except for the AKD1_agar_H sample stored under ambient conditions. b) Photo of a water drop on the AKD10_CNF coating. c) WCA of reference polysaccharide–only, AKD–only, and CFM sample.

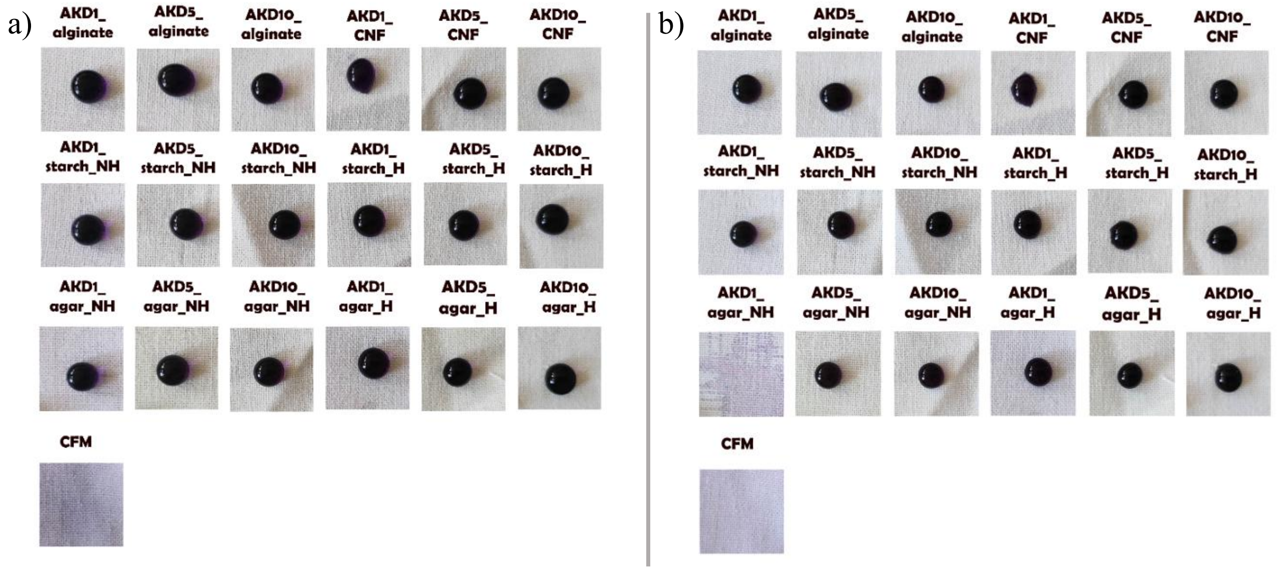

Figure S7. (a) Water absorption behavior immediately after water deposition, showing rapid penetration only for the untreated cotton fabric membrane (CFM). (b) Water absorption behavior after 1h, demonstrating that water droplets remain stable on all coated surfaces except the AKD1_agar_NH sample.

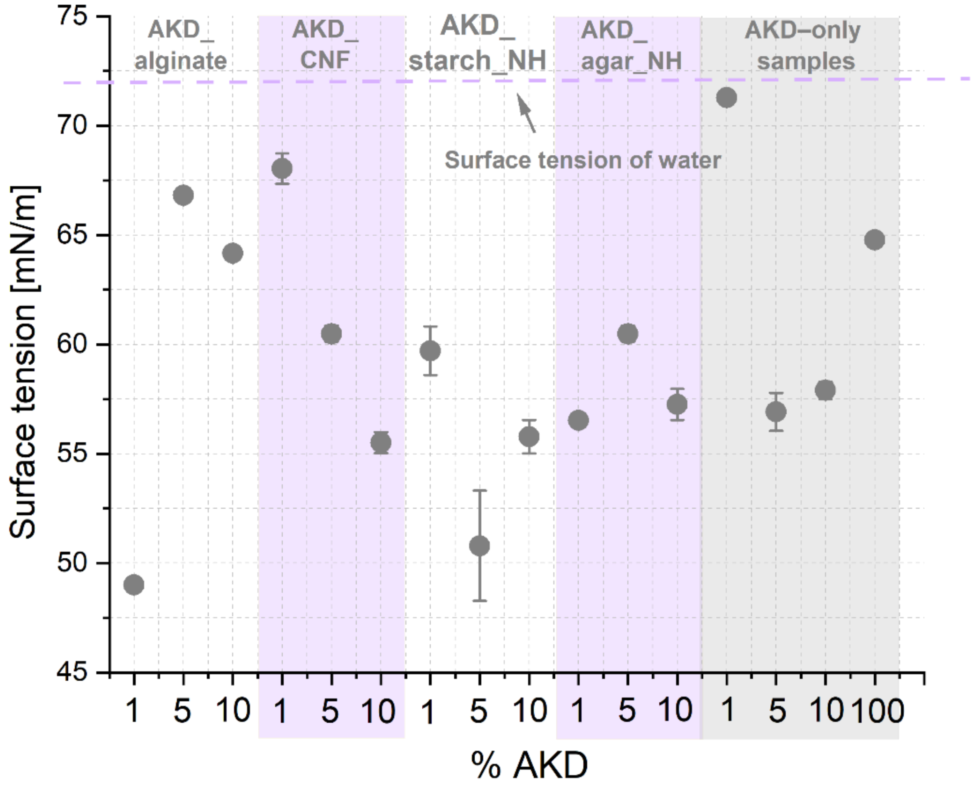

Figure S8. Surface tension measurements of the coating dispersions show that AKD reduces the surface tension. At AKD1, this effect is negligible, indicating that polysaccharides also contribute to reducing surface tension, thereby enhancing liquid spreading and supporting their templating role during coating formation.

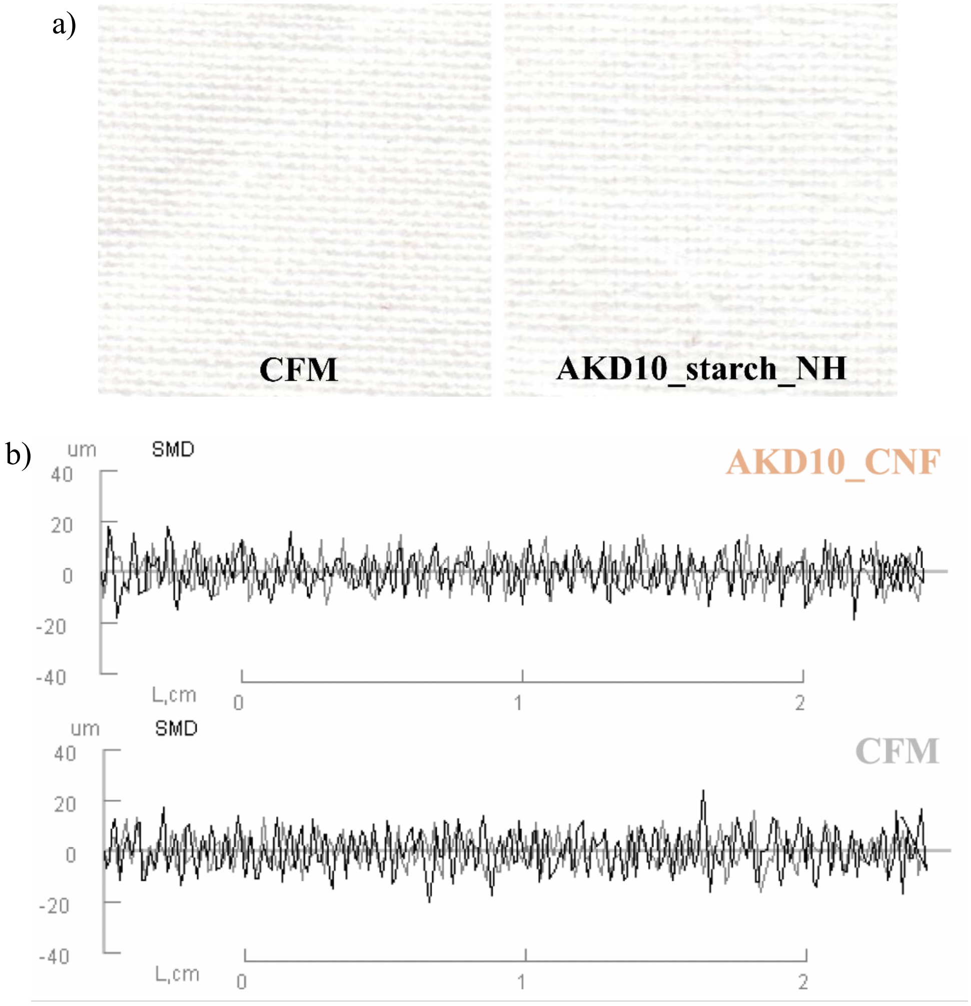

Figure S9. (a) Surface scan images for cellulosic fiber material (CFM) and AKD10_starch_NH show no visible differences between untreated and coated samples. (b) SMD plots of CFM and AKD10_CNF sample confirm that the geometrical surface roughness remains almost unchanged after coating deposition.

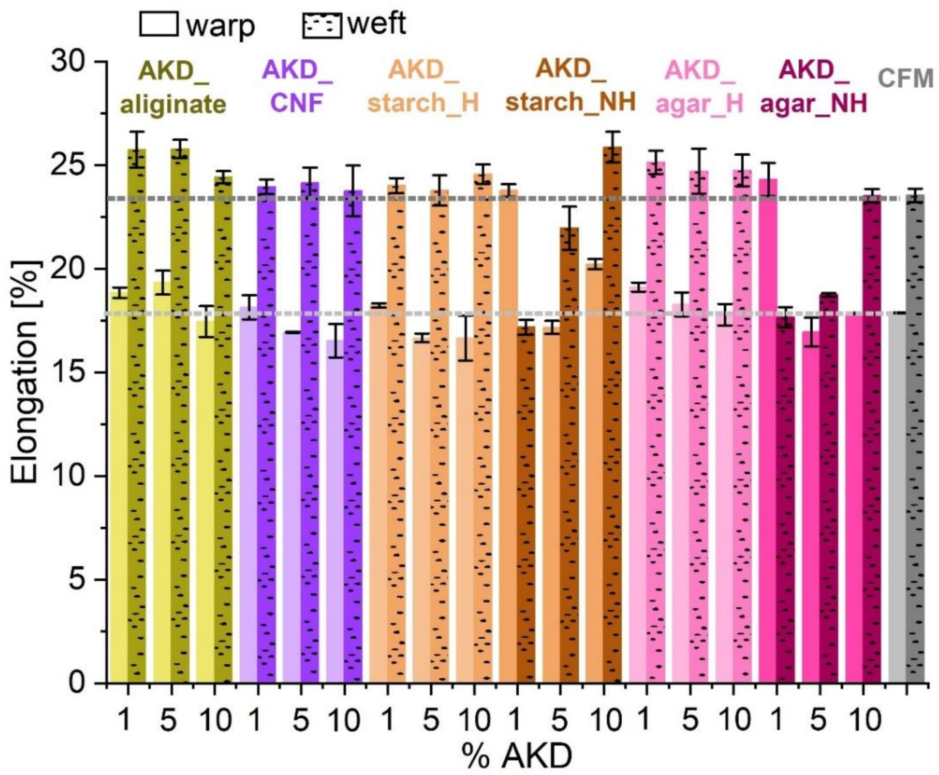

Figure S10. Elongation results of AKD–polysaccharide samples and reference cellulosic fiber material (CFM) showing that lower AKD concentrations generally allow greater elongation, whereas higher concentrations reduce elongation or maintain values close to those of untreated CFM.

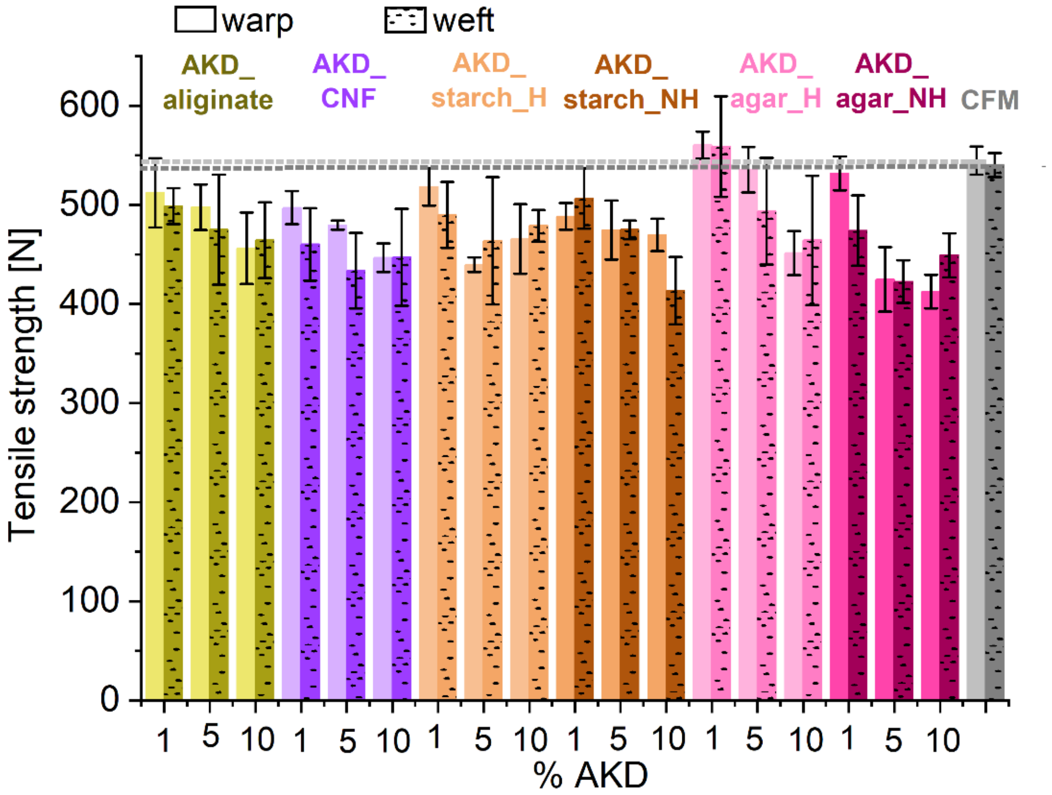

Figure S11. Tensile strength results for AKD–polysaccharide samples and reference cellulosic fiber material (CFM) showing a decrease for most samples compared with the untreated cellulosic fiber material.

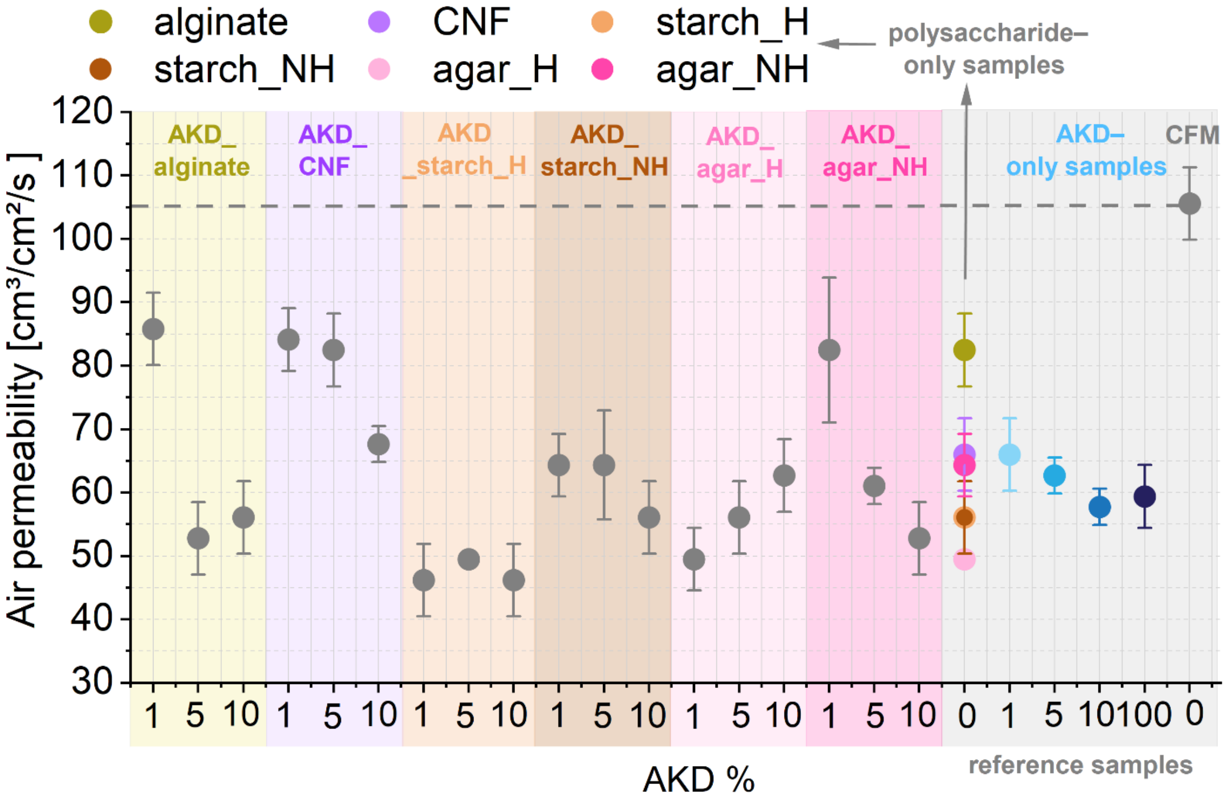

Figure S12. Air permeability results of AKD–polysaccharide coatings, reference polysaccharide–only and AKD–only samples showing reduced air permeability for all coating samples compared to untreated cellulosic fiber material (CFM).

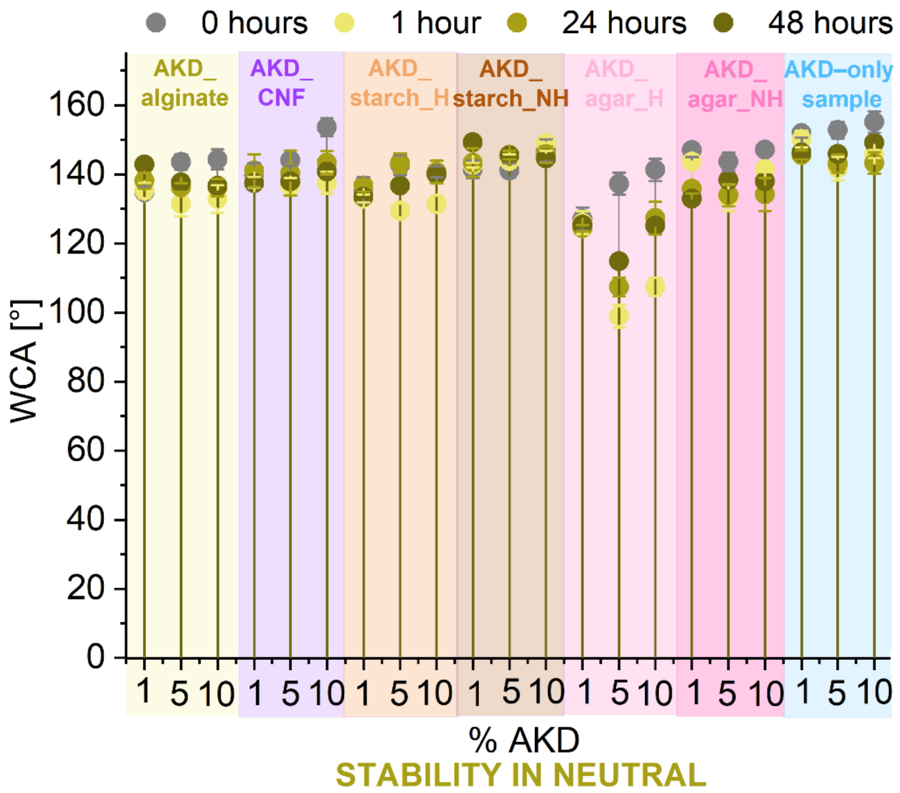

Figure S13. WCA of AKD–polysaccharide and AKD–only samples after exposure to neutral conditions, indicating relatively good stability even after 48 hours of exposure.

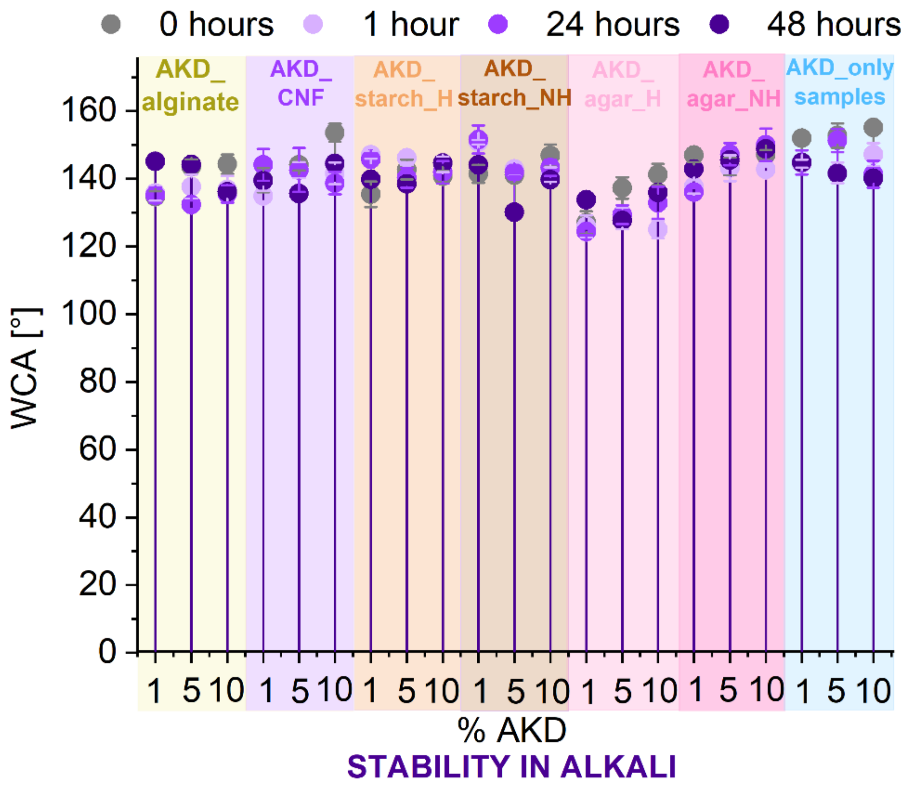

Figure S14. WCA of AKD–polysaccharide and AKD–only samples after exposure to alkali conditions, indicating relatively good stability even after 48 hours of exposure.

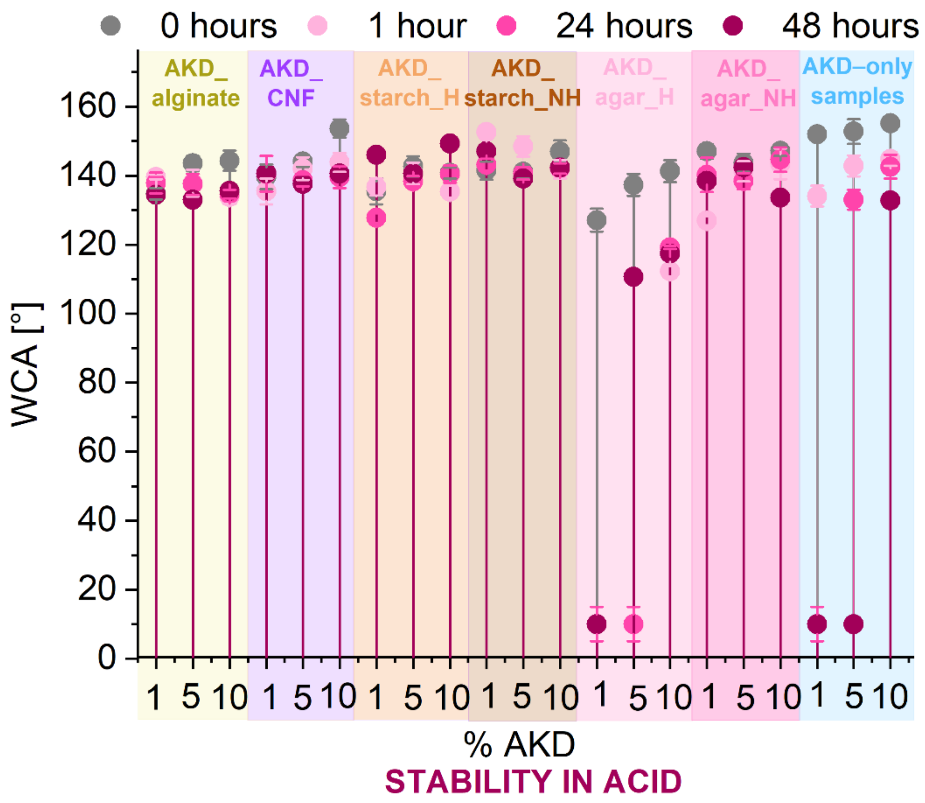

Figure S15. WCA of AKD–polysaccharide and AKD–only samples after exposure to acid conditions, indicating relatively good stability for most samples even after 48 hours of exposure, with reduced stability observed for AKD1_agar_H, AKD5_H, AKD1, and AKD5.

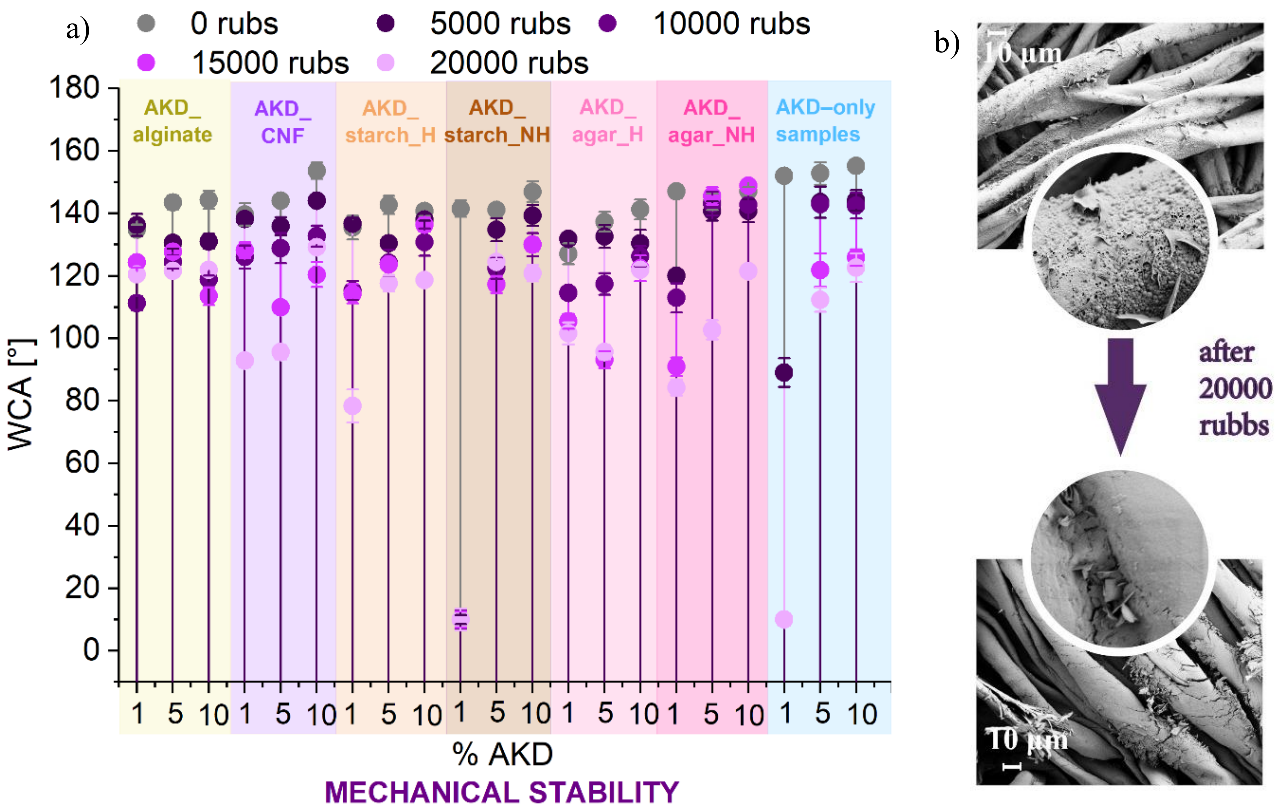

Figure S16. (a) WCA after 20 000 rubbing cycles for AKD–polysaccharide coating and AKD–only samples, demonstrating adequate mechanical stability and improved durability of AKD coatings when combined with selected polysaccharides. (b) SEM images of the AKD10_CNF sample after 20 000 rubbing cycles, showing partial removal of the coating from the surface; however, flake-like AKD structures remain preserved.

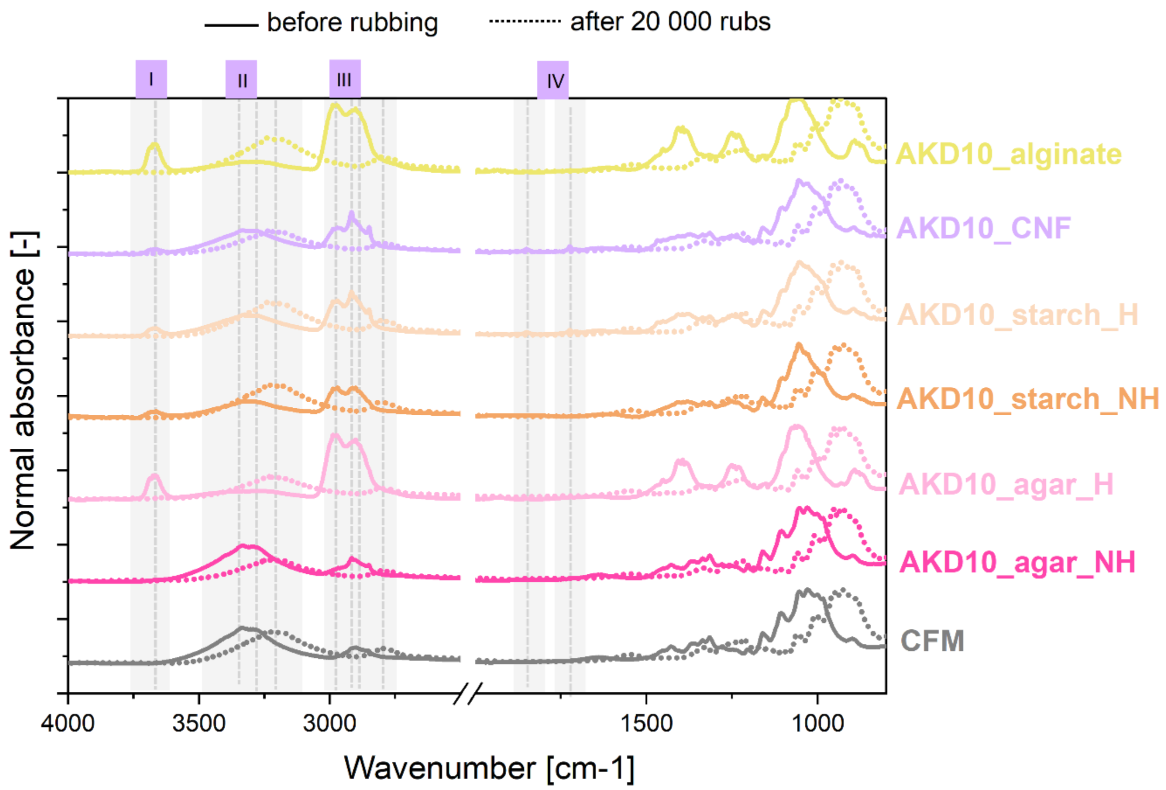

Figure S17. Comparison of FTIR spectra of CFM and AKD10_polysaccharide samples before and after 20 000 rubbing cycles, showing changes in C–H and O–H stretching regions and the fingerprint region.

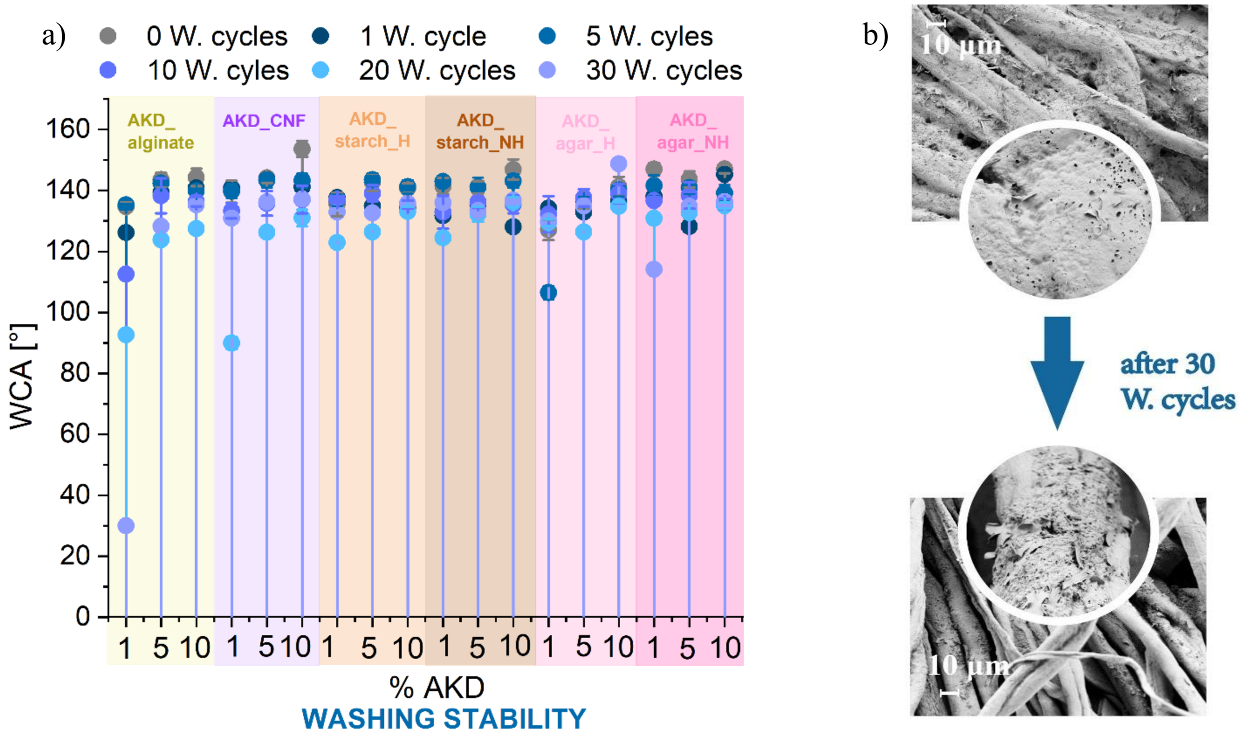

Figure S18. (a) WCA of AKD–polysaccharide samples after washing tests, showing good stability after 30 washing cycles for most coatings, except some AKD1_polysaccharide samples. (b) SEM images of the AKD10_agar_H sample before and after 30 washing cycles, showing removal of the agar film after washing and increased exposure of AKD domains.

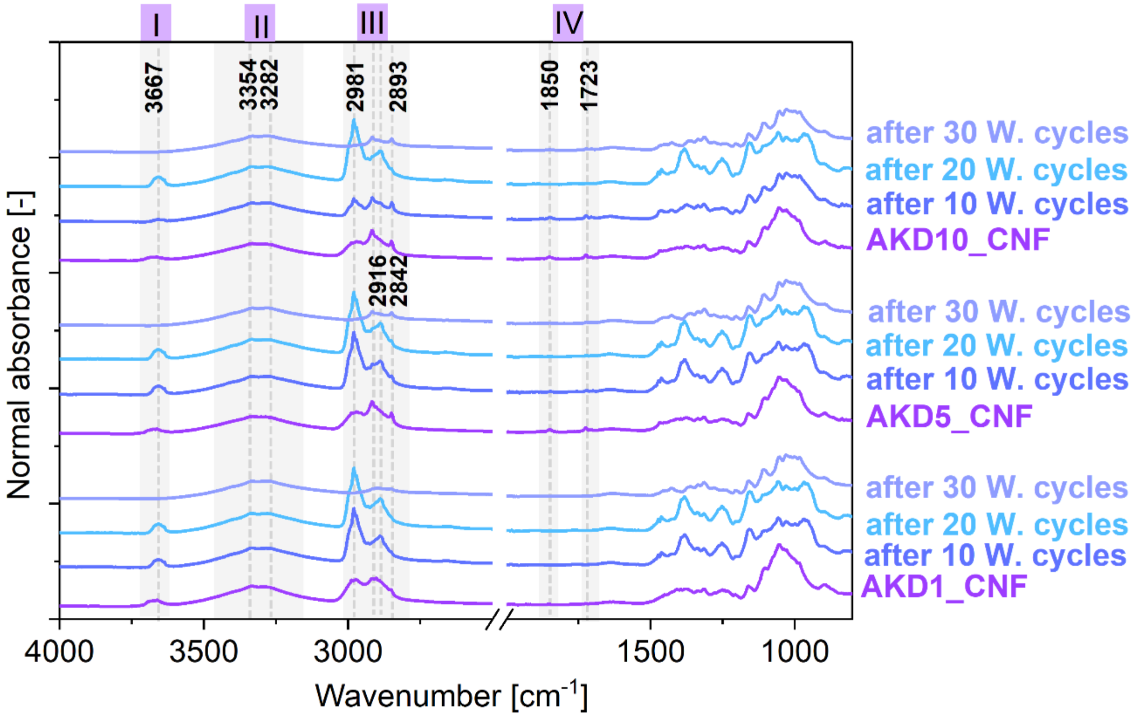

Figure S19. FTIR spectra of the AKD_CNF samples show an increase in C–H stretching intensity after 10 and 20 washing cycles, followed by a pronounced decrease after 30 washing cycles.

Table S1. Summary of reported AKD–only and AKD–polysaccharide coatings on cellulosic substrates, detailing substrate type, coating formulation, hydrophobic performance, curing conditions, durability, physical properties, and any additional functionalities.

**Reference**	**Substrate**	**Formulation**	**Hydrophobic performance**	**Curing time & temperature**	**Durablity**	**Physical properties and multifunctionality**
Onodera et al.[1] (2025)	Cotton knitted fabric	Weakly cationic AKD dispersion (o,5% of dry fabric weight)	WCA=150°, water absorbtion time > 1800s.	10 min at 120 °C	Washing durability (30 × JIS L 1930 FC4M laundry cycles): AKD related compound ↓ 0,06%, water absorbtion time > 1800s; $CHCl_3$ extraction:AKD related compound ↓ 0,01%, water absorbtion time ~ 2000s; hot-water extraction: AKD related compound ↓ 0,24%, water absorbtion time > 1800s; tween 80 extraction: AKD related compound ↓ 0,03%, water absorbtion time ~ 38s;	Stiffness: ↑ 0.04N<br>.
Thananukul et al.[2] (2025)	Cotton fabric	Water-based AKD nanopartic	WCA=137 ± 3°	5 h at 110 °C	Stable after 20 washing cycles and 14 days UV exposure, WCA = 136 ±	Bifunctional: antibacterial (94.7% S. aureus,

		les (182.5 ± 1.0 nm);			5° (after 20 washes)	70.2% E. coli).
Wang et al.[3] (2023)	Chitosan (emulsifier), TiO_2 (possible second emulsiofier) acetic-acid solution, AKD	Cellulosic filter paper	154°	45°C (optimal)	N/A	Oil–water separation efficiency over 93%.
Nechita et al.[4] (2022)	Food packaging paper	Xylan hemicellulose, AKD: (0.2%, 0.5%, 1.0%, 1.5%)	92° (1.5% AKD)	~110 °C	N/A	For 1,5 AKD: WVTR ↓ 35% vs base paper; water absorption ↓ 29%, Wet-tensile index: ↑ 6% bursting strength: ↑13% increase; air permeability: ↓ 66 %.
Oh et al.[5] (2022)	CNF sheet	AKD wax solution (5–30 % w/w in toluene)	120°	5 min wet-press, 95 °C drum-dry	N/A	WVTR: ↓9%
Adenekan & Hutton-Prager[6] (2019)	Whatman filter paper	AKD/n-heptane, CO_2 impregnation	140 ± 5°	Ambient temperature (no thermal cure)	sticky hydrophobicity >140 days; CAH 17 ± 5°	N/A
Liu et al.[7] (2023)	Packaging paper (APMP pulp)	AKD, guar gel (0.25%, 0.5%, 0.75%, 1.0%, 1.25%),	WCA=129°	30 min at 105°C	N/A	N/A

		sodium tetraborate				
Mazega et al.[8] (2022)	eucalyptus kraft paper	CNF, AKD, Alkenyl succinic anhydride (ASA)	121°	air-drying	N/A	Air-permeability ≈ 9 s · 100 m L^{-1} (no significant change), internal-bond strength increase ↑0.2 kJ m^{-2}

References

(1) Onodera, S.; Tanaka, C.; Isogai, A. Water Repellency of Cotton Knitted Fabrics Treated with Alkyl Ketene Dimers. *Cellulose* **2025**. https://doi.org/10.1007/s10570-025-06390-5.

(2) Thananukul, K.; Kaewsaneha, C.; Roeurn, B.; Opaprakasit, M.; Kyaw, Z. W.; Khimlek, W.; Opaprakasit, P. Bifunctional Water-Based Alkyl Ketene Dimer Nanoparticles for Fabricating Cotton Fabrics with Hydrophobicity and Antimicrobial Properties. *ACS Appl. Nano Mater.* **2025**. https://doi.org/10.1021/acsanm.5c00586.

(3) Wang, Y.; Huang, Y.; Zhong, J.; Yu, C. Superhydrophobic Coatings on Cellulose-Based Materials with Alkyl Ketene Dimer Pickering Emulsion: Fabrication and Properties. *Coatings* **2023**, *13* (11). https://doi.org/10.3390/coatings13111829.

(4) Nechita, P.; Roman, M.; Cantaragiu Ceoromila, A.; Dediu Botezatu, A. V. Improving Barrier Properties of Xylan-Coated Food Packaging Papers with Alkyl Ketene Dimer. *Sustainability (Switzerland)* **2022**, *14* (23). https://doi.org/10.3390/su142316255.

(5) Oh, Y.; Park, S. Y.; Yook, S.; Shin, H.; Lee, H. L.; Youn, H. J. A Waterproof Cellulose Nanofibril Sheet Prepared by the Deposition of an Alkyl Ketene Dimer on a Controlled

Porous Structure. *Cellulose* **2022**, *29* (12), 6645–6657. https://doi.org/10.1007/s10570-022-04701-8.

(6) Adenekan, K.; Hutton-Prager, B. Sticky Hydrophobic Behavior of Cellulose Substrates Impregnated with Alkyl Ketene Dimer (AKD) via Sub- and Supercritical Carbon Dioxide. *Colloids Surf. A Physicochem. Eng. Asp.* **2019**, *560*, 154–163. https://doi.org/10.1016/j.colsurfa.2018.09.073.

(7) Liu, X.; Li, Y.; Wang, H.; Song, Z.; Tan, C.; Li, G.; Yu, D.; Liu, W. AKD Emulsions Stabilized by Guar Gel: A Highly Efficient Agent to Improve the Hydrophobicity of Cellulose Paper. *Polymers (Basel).* **2023**, *15* (24). https://doi.org/10.3390/polym15244669.

(8) Mazega, A.; Tarrés, Q.; Aguado, R.; Pèlach, M. À.; Mutjé, P.; Ferreira, P. J. T.; Delgado-Aguilar, M. Improving the Barrier Properties of Paper to Moisture, Air, and Grease with Nanocellulose-Based Coating Suspensions. *Nanomaterials* **2022**, *12* (20). https://doi.org/10.3390/nano12203675.
